# Profiling of the muscle-specific dystroglycan interactome reveals the role of Hippo signaling in muscular dystrophy and age-dependent muscle atrophy

**DOI:** 10.1186/s12916-019-1478-3

**Published:** 2020-01-21

**Authors:** Andriy S. Yatsenko, Mariya M. Kucherenko, Yuanbin Xie, Dina Aweida, Henning Urlaub, Renate J. Scheibe, Shenhav Cohen, Halyna R. Shcherbata

**Affiliations:** 1 Gene Expression and Signaling Group, Institute of Cell Biochemistry, Hannover Medical School, Carl-Neuberg-Strasse 1, 30625 Hannover, Germany; 2 Max Planck Research Group of Gene Expression and Signaling, Max Planck Institute for Biophysical Chemistry, Am Fassberg 11, 37077 Göttingen, Germany; 3 Present Address: Department of Cardiothoracic and Vascular Surgery, German Heart Center Berlin, Augustenburger Platz 1, 13353 Berlin, Germany; 4 Institute of Physiology, Charité - University Medicine Berlin, Charitéplatz 1, 10117 Berlin, Germany; 5 Present Address: University Medical Center, Centre for Anatomy, Institute of Neuroanatomy, Georg-August-University Göttingen, Kreuzbergring 36, 37075 Göttingen, Germany; 6 Faculty of Biology, Technion, 32000 Haifa, Israel; 7 Bioanalytical Mass Spectrometry Research Group, Max Planck Institute for Biophysical Chemistry, Am Fassberg 11, 37077 Göttingen, Germany; 8 Bioanalytics Institute for Clinical Chemistry, University Medical Center Goettingen, Robert Koch Strasse 40, 37075 Göttingen, Germany

**Keywords:** Dystroglycan, Hippo kinase signaling, Kibra, Yorkie, Proteomics, Muscle degeneration, Muscle atrophy, Muscular dystrophies

## Abstract

**Background:**

Dystroglycanopathies are a group of inherited disorders characterized by vast clinical and genetic heterogeneity and caused by abnormal functioning of the ECM receptor dystroglycan (Dg). Remarkably, among many cases of diagnosed dystroglycanopathies, only a small fraction can be linked directly to mutations in Dg or its regulatory enzymes, implying the involvement of other, not-yet-characterized, Dg-regulating factors. To advance disease diagnostics and develop new treatment strategies, new approaches to find dystroglycanopathy-related factors should be considered. The Dg complex is highly evolutionarily conserved; therefore, model genetic organisms provide excellent systems to address this challenge. In particular, *Drosophila* is amenable to experiments not feasible in any other system, allowing original insights about the functional interactors of the Dg complex.

**Methods:**

To identify new players contributing to dystroglycanopathies, we used *Drosophila* as a genetic muscular dystrophy model. Using mass spectrometry, we searched for muscle-specific Dg interactors. Next, in silico analyses allowed us to determine their association with diseases and pathological conditions in humans. Using immunohistochemical, biochemical, and genetic interaction approaches followed by the detailed analysis of the muscle tissue architecture, we verified Dg interaction with some of the discovered factors. Analyses of mouse muscles and myocytes were used to test if interactions are conserved in vertebrates.

**Results:**

The muscle-specific Dg complexome revealed novel components that influence the efficiency of Dg function in the muscles. We identified the closest human homologs for Dg-interacting partners, determined their significant enrichment in disease-associations, and verified some of the newly identified Dg interactions. We found that Dg associates with two components of the mechanosignaling Hippo pathway: the WW domain-containing proteins Kibra and Yorkie. Importantly, this conserved interaction manages adult muscle size and integrity.

**Conclusions:**

The results presented in this study provide a new list of muscle-specific Dg interactors, further analysis of which could aid not only in the diagnosis of muscular dystrophies, but also in the development of new therapeutics. To regulate muscle fitness during aging and disease, Dg associates with Kibra and Yorkie and acts as a transmembrane Hippo signaling receptor that transmits extracellular information to intracellular signaling cascades, regulating muscle gene expression.

## Background

**Fig. 1:**
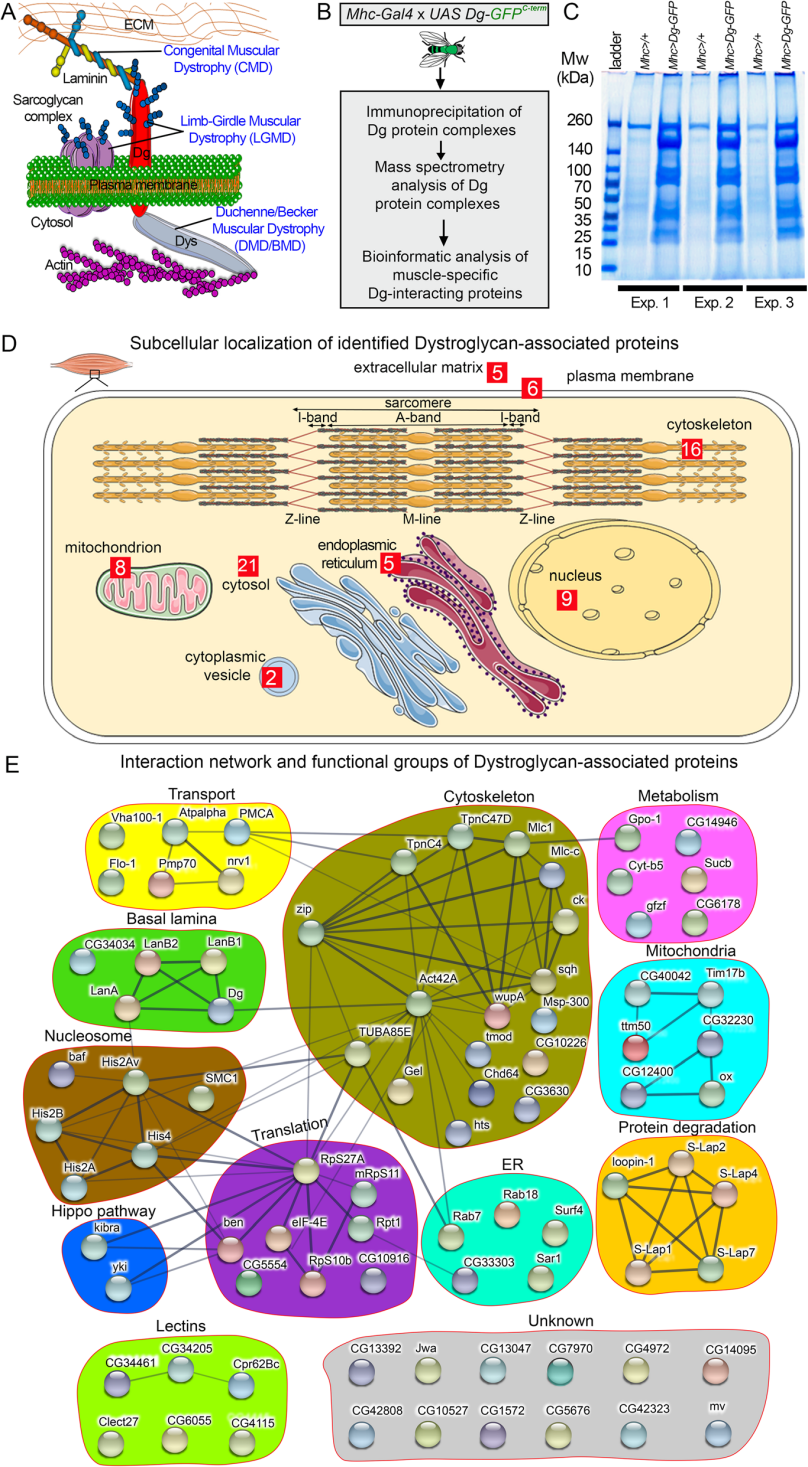
Identification of dystroglycan-associated proteins. **a** Schematic of *Drosophila* dystrophin glycoprotein complex (DGC). The transmembrane protein dystroglycan (Dg) is a major component of the DGC. Its C-terminal end binds the cytoplasmic protein dystrophin (Dys), while the heavily glycosylated N-terminus is associated with extracellular matrix (ECM) proteins. In humans, perturbed DGC function results in various types of neuromuscular disorders such as congenital muscular dystrophy (CMD), limb-girdle muscular dystrophy (LGMD), and Duchenne/Becker muscular dystrophy (DMD/BMD). **b** The pipeline used to identify muscle-specific Dg-associated proteins. Firstly, we generated transgenic animals expressing in the muscle tissue the full-length Dg tagged by the GFP at the C-terminal end (*Mhc-Gal4/+*, *UAS Dg::GFP/+*, abbreviated *Mhc>Dg)*. Secondly, tissue lysates were subjected to co-immunoprecipitation procedure with GFP-Trap beads. Thirdly, immunoprecipitated Dg protein complexes were analyzed by mass spectrometry in order to identify Dg-interacting proteins. Lastly, revealed Dg-associated components were bioinformatically analyzed. **c** Coomassie Blue-stained gel confirms increased protein levels in the samples with muscle-specific Dg overexpression. Experiments were performed in triplicate. As a control, the driver line crossed to a wild-type line was used (*Mhc-Gal4/OregonR*, abbreviated *Mhc>/+).* **d** Schematic of muscle cell with subcellular compartments (plasma membrane, cytoskeleton, mitochondria, cytoplasmic vesicles, nucleus, endoplasmic reticulum, and Golgi apparatus). The scheme was created with the SERVIER Medical Art. Subcellular localization of the identified Dg-associated proteins. **e** Protein association network for Dg-interacting proteins clustered in functional groups, among which are structural components of the basal lamina, proteins with functions in muscle cell cytoskeleton, cellular transport regulators, mitochondria-associated proteins, factors regulating protein biosynthesis and degradation, cellular metabolism, nucleosome components, and proteins from the Hippo signaling pathway. Nodes represent proteins, lines and dashed lines connect associated proteins, and line thickness shows the confidence of association. Markov clustering algorithm identifies several protein complexes (thick blue lines) within basal lamina, cytoskeleton, protein degradation, mitochondria, and nucleosome groups

In higher organisms, the muscles express specialized protein complexes, e.g., the dystrophin glycoprotein complex (DGC, Fig. 1a), which permits transmission of forces from the extracellular matrix (ECM) to the inside of the cell and reduces mechanical stress placed on the plasma membrane, thus shielding the fragile sarcolemma. DGC dysfunctions cause the development of the pathological states commonly called muscular dystrophies (MDs) in humans, which is a large group of genetic diseases associated with muscle weakening and loss. Among the DGC-associated muscular dystrophies are Duchenne/Becker muscular dystrophies (DMD/BMD), resulting from mutations in the cytoplasmic protein dystrophin that connects actin cytoskeleton to the muscle membrane; limb-girdle muscular dystrophies (LGMDs), caused by the dysfunction of the membrane-associated dystroglycan and sarcoglycan multiprotein complexes; and congenital muscular dystrophies (CMDs), triggered by the deregulation of dystroglycan and the ECM protein laminin (Fig. 1a). Due to the high rates of these deadly diseases, intensive research has been directed towards understanding the role of the DGC in providing mechanical stability to the muscle sarcolemma. However, it has been shown that the DGC not only offers strength to the muscle but also is capable of mediating intra- and extracellular signaling [1–3].

Since dystroglycanopathies encompass significant clinical, pathological, and genetic heterogeneity, their proper diagnosis is challenging. For example, primary dystroglycanopathies result from a mutation in the *Dg* gene per se (*DAG1* in vertebrates), while deficiencies in 17 genes encoding the enzymes involved in Dg glycosylation have been identified and associated with secondary dystroglycanopathies [4, 5]. Currently, the diagnosis of these fatal neuromuscular disorders is implemented on the basis of (i) clinical and biological features associated with limb-girdle muscular dystrophy, lissencephaly type II, and congenital muscle dystrophy and (ii) elevated levels of a sensitive parameter of muscle damage, creatine kinase. These steps are followed by (iii) gene sequencing to identify mutations in the abovementioned 18 genes [4–7]. Despite recent advances, the efficiency of accurate genetic and molecular diagnoses is rather low—only 36% in children and 22% in adults [4, 8]. It was experimentally validated that in the sarcolemma of muscle fibers, Dg arranges various protein complexes even in the absence of both dystrophin and utrophin [9], supporting the idea about a broader role for Dg, apart from being a core DGC component. To advance the disease diagnostics and develop new treatment strategies, it is essential to identify novel components that influence the efficiency of Dg function in the muscles. In addition to genetic causes, multiple other internal and external stress factors trigger muscle wasting; for example, aging is often associated with sarcopenia, nutritional deficit, and cancer leading to cachexia, while sleep deprivation and hormonal imbalance cause muscle atrophy [10–12]. Unfortunately, nutritional supplements and physical exercises have only very limited abilities to ameliorate these conditions and restore muscle mass and function. Therefore, a search for novel components modulating muscle maintenance under stress is important for improving active life expectancy in older people or cancer patients.

Due to the fact that the Dg complex is highly evolutionarily conserved, model genetic organisms including *Drosophila* provide an excellent system to dissect the functions of Dg itself and get insight into its cellular lifecycle and involvement in different signaling pathways. Previously, we have shown that *Drosophila* can serve as a great system to study MDs, since Dg deficiency leads to a range of muscular and neuronal dysfunctions [13]. In addition, Dg gene structure is remarkably conserved [14]. *Drosophila* Dg has functions distinct from Dys and, apart from its role in muscle maintenance, is involved in the regulation of energy homeostasis, photoreceptor differentiation, neuromuscular junction establishment, and cellular polarity [15–20]. Precise control of Dg expression during development is critical; its elevation can be as dramatic as its deficiency. At the post-transcriptional level, Dg is subject to miRNA-based regulation in various tissues [3, 21]. Moreover, the DGC can regulate the expression of miRNA genes, in particular via a Dg-Dys-Syn1 signaling cascade [3, 22]. The DGC-dependent effect on the miRNA profile is linked to stress, and there is a correlation between miRNAs regulated by either DGC or stress conditions [22]. Furthermore, stress causes muscle wasting even in healthy *Drosophila* animals, while DGC-mutant muscles are already compromised and more susceptible to stress [18]. Together, these data imply that the transmembrane protein Dg has a potential role of sensing extracellular stimuli (stressors) and transmitting signals to the cell, governing cellular stress responses. With regard to the muscle tissue that continuously withstands vigor of contractions, it is likely that Dg not only serves as a physical anchor between the ECM and the cytoskeleton, but also plays a role in transducing mechanical stresses sensed from the ECM to the intracellular signaling pathways controlling gene expression. This function is extremely important, since throughout life, muscle cells are constantly exposed to mechanical forces. Although these forces are important regulators of normal cell function, muscle cell membrane rupture occurs frequently even under physiological conditions [23], which is most prevalent in the skeletal and cardiac muscles. It results in the influx of extracellular Ca^2+^ and the loss of cytoplasmic ingredients. This disturbs the maintenance of the balance between the cytoplasm and the extracellular space and causes contraction defects and/or cell death [24, 25].

Moreover, in muscle cells, mechanical forces induce a tension-driven mechanism for the self-organization of myofibrils during development [26]. One of the most famous mechanotransducing pathways is the evolutionarily conserved Hippo signaling pathway. Yap (Yes-associated protein), a mammalian homolog of *Drosophila* Yorkie (Yki), is a nuclear factor of the Hippo kinase signal transduction pathway that relays on mechanical signals exerted by the ECM stiffness, cell geometry, and cytoskeletal tension to mediate cellular mechanoresponses [27]. The key to this pathway is a kinase cascade in which Hippo phosphorylates and activates Warts, which in turn phosphorylates and inactivates Yap/Yki [28]. Yap/Yki together with TEAD/TEF transcription factors regulate the expression of genes that promote cell proliferation, prevent apoptosis, or control innate immunity [29–31]. Importantly in mammalian adult skeletal muscles, Yap has been shown to positively regulate skeletal muscle mass and protein synthesis following injury and upon neurogenic muscle atrophy [32] and have differential expression in healthy and dystrophic muscles [33, 34]. Recent studies revealed the connection between the DGC and the Hippo pathway in the mouse heart; however, how this interaction influences Hippo and DGC signaling is not understood. For example, in the developing, proliferative mouse heart, Yap regulates the expression of the *Sgcd* gene encoding sarcoglycan, which is a part of the DGC [35]; while in the adult heart, Dg (DAG1) directly binds and sequesters phosphorylated Yap to inhibit cardiomyocyte proliferation [36]. While the previous data strongly support the idea that the key DGC component, Dg, plays a role in mechanotransduction and clearly demonstrate that Hippo signaling is a key pathway responding to mechanical stress, the functional link between the DGC and Hippo kinase signaling in the maintenance of skeletal muscle tissue integrity and architecture remains unclear.

In this study, we analyzed the *Drosophila* Dg interactome to identify novel muscle-specific components associated with the DGC using mass spectrometry. We experimentally validated the interaction and co-localization of Dg with several identified candidates. In particular, we verified Dg interactions in different types of larval and adult muscles with LamininA subunits and several other muscle cytoskeleton proteins. Importantly, we found Hippo protein kinase signaling pathway components to physically and genetically interact with Dg in adult fast-twitch oxidative muscles and revealed the requirement for Hippo signaling in control of muscle size and integrity during aging. Based on our findings, we propose that Dg associates with Kibra (Kbr) and Yki in adult muscles and possibly acts as a mechanosensing receptor for the Hippo signaling pathway, modulating its function. Thus, our data show a conserved role of Dg in mediating Hippo signaling in muscle cells, which manages muscle growth and regeneration in an age-dependent manner.

## Methods

### *Drosophila* stocks

*Drosophila* stocks and crosses were maintained at standard corn meal, yeast, glucose agar medium at 25 °C under 12-h:12-h light:dark cycles.

To co-immunoprecipitate Dg from the muscle tissue, the transgenic animals bearing C-terminally GFP-tagged Dg construct *UAS-Dg::GFP* [17] were crossed to *Mhc-Gal4 (BL #55132)* driver line allowing for the muscle-specific Dg expression, which resulted in sixfold increase in Dg levels (see Additional file 1: Data S1). As a control, the driver line crossed to a wild-type line was used (*Mhc-Gal4/OregonR*, abbreviated *Mhc>/+*) or to *UAS-CD8::GFP (BL #5137)*.

To address the co-localization of Dg with other identified muscle components, the collection of strains expressing GFP-tagged proteins [37] was used: *LanA-GFP (VDRC #318155)*, *LanB1::GFP (VDRC #318180)*, *hts::GFP (VDRC #318163)*, *wupA::GFP (VDRC #318278)*, and *yki::GFP (VDRC #318237).*

To downregulate or overexpress the studied proteins in the muscles, the following transgenic constructs were used: *UAS-dsDg30A* (*Dg*^*RNAi*^) [13], *UAS-ykiRNAi* (*yki*^*RNAi*^, *BL34067*), *UAS-kbrRNAi* (*kbr*^*RNAi*^, *BL28683*), *UAS-yki (BL28816)*, and *UAS-kbr (VDRC 100705).*

To identify genetic interactions of Dg with Hippo pathway components in the wings and muscles, the Dg loss-of-function mutants *Dg*^*O55*^, *Dg*^*O43*^, and/or *Dg*^*O86*^ [38] were crossed with the following Hippo pathway mutants: *FRT82B kbr*^*del (483-5860)*^*/TM6* (*kbr*^*del*^), *FRT42D hpo*^*42-47*^*/SM6* (gifts from Wu-Min Deng), and *FRT42D yki*^*B5*^*/CyO* (a gift from DuoJia Pan). The progeny heterozygous for *Dg* and a tested mutant allele were collected for indirect flight muscle (IFM) analyses.

For muscle analysis, several conditions were applied. Hatched adults, kept for 7 days at normal laboratory conditions, were considered as young animals, while adults maintained for 28–30 days at the same conditions were reflected as aged.

### Co-immunoprecipitation and western blot analysis in *Drosophila*

Whole lysates for general co-immunoprecipitation (co-IP) were prepared from approximately 1-week-old flies that were homogenized with VWR Disposable Pellet Mixers and lysed in RIPA buffer containing 50 mM Tris-HCl (pH 7.5), 125 mM NaCl, 5% glycerol, 0.5% NP40, 0.25% Na-deoxycholate, 1.5 mM MgCl2, 1 mM dithiothreitol, 25 mM NaF, 1 mM Na3VO4, 1 mM EDTA, 2 mM EGTA, and protease inhibitors. Samples were then centrifuged at 15,000*g* for 15 min at 4 °C, and then 1.3 mg of supernatants was immunoprecipitated with GFP-Trap beads (ChromoTek) following the manufacturer’s instructions. Four percent of total protein extracts used for immunoprecipitation were loaded as input. The presence of immunocomplexes was analyzed via SDS-PAGE and western blotting as described in [39]. The following antibodies were used: rabbit anti-Kibra (1:1000, a gift from Wu-Min Deng), rabbit anti-Laminin (1:1000, Abcam ab47651), mouse anti-Hts (also called Adducin, 1:1000, DSHB), and chicken anti-GFP (1:5000, Abcam ab13970).

For the co-IP coupled to mass spectrometry, whole lysates were prepared from 1-week-old flies harvested by snap freezing in liquid nitrogen. Three biological replicates of 3–4 mg of both control (*Mhc-Gal4/+*) and experimental flies (*Mhc-Gal4/UAS-Dg::GFP*) were homogenized and lysed by grinding in 3 ml of the abovementioned RIPA buffer in a mortar with pestle using liquid nitrogen. Lysates were cleared by two centrifuging steps once for 15 min and once for 10 min at 15000*g* at 4 °C. Next, 36 mg of protein extract was added to 26 μl of GFP-Trap beads in 2 × 1.5 ml micro-centrifuge tubes and incubated rotating at 4 °C overnight. To collect the beads, lysates were centrifuged at 2500*g* for 2 min at 4 °C. The beads were washed six times with ice-cold RIPA lysis buffer and were finally eluted with 40 μl of warm 2× sample buffer (NuPAGE LDS Sample Buffer, Novex). The eluates were analyzed by mass spectrometry after SDS-PAGE.

### Coomassie Brilliant Blue staining

The Coomassie Brilliant Blue (CBB) staining was performed as previously described [40]. In brief, CBB G-250 is dissolved in double-distilled water in a concentration of 60–80 mg/l, and 35 mM HCl is added as the only other compound in the staining solution. The gel from SDS-PAGE was rinsed with double-distilled water and incubated in CBB staining solution overnight at room temperature with gentle shaking. Next, the stained gel was distained by washing with double-distilled water.

### Gel electrophoresis, in-gel digestion, and mass spectrometry (LC-MS/MS)

Proteins were separated by one-dimensional SDS-PAGE (4–12% NuPAGE Bis-Tris Gel, Invitrogen) and stained with Coomassie Blue G-250 (Fluka). The complete gel lanes were cut into 23 equally sized slices. Proteins were digested as described previously [41]. Briefly, proteins were reduced with 10 mM DTT for 50 min at 50 °C, afterwards alkylated with 55 mM iodoacetamide for 20 min at 26 °C. In-gel digestion was performed with Lys-C (Roche Applied Science) overnight. Extracted peptides from gel slices were loaded onto the in-house packed C18 trap column (ReproSil-Pur 120 C18-AQ, 5 μm, Dr. Maisch GmbH; 20 × 0.100 mm) at a flow rate of 5 μl/min loading buffer (2% acetonitrile, 0.1% formic acid). Peptides were separated on the analytical column (ReproSil-Pur 120 C18-AQ, 3 μm, Dr. Maisch GmbH; 200 × 0.050 mm, packed in-house into a PF360-75-15-N picofrit capillary, New Objective) with a 90-min linear gradient from 5 to 40% acetonitrile containing 0.1% formic acid at a flow rate of 300 nL min^−1^ using nanoflow liquid chromatography system (EASY n-LC 1000, Thermo Scientific) coupled to hybrid quadrupole-Orbitrap (Q Exactive, Thermo Scientific). The mass spectrometer was operated in data-dependent acquisition mode where survey scans acquired from *m*/*z* 350–1600 in the Orbitrap at resolution settings of 70,000 FWHM at *m*/*z* 200 at a target value of 1 × 10e6. Up to 15 most abundant precursor ions with charge states 2+ or more were sequentially isolated and fragmented with higher collision-induced dissociation (HCD) with normalized collision energy of 28. Dynamic exclusion was set to 18 s to avoid repeating the sequencing of the peptides. It is important to stress that current mass spectrometry techniques are quite limited; depending on the method and tissue only 10–30% of all known *Drosophila* proteins can be detected, most of which are highly expressed, housekeeping genes [42]. The muscle in particular is a highly specialized tissue that expresses very high levels of distinct structural proteins, which makes it challenging for mass spectrometry analysis. For example, in our experiment, myosin heavy chain represented ~ 30% of all hits, as it is the most highly expressed muscle protein (Additional file 1: Data S1). This could have prevented the identification of some proteins that are expressed at significantly lower levels.

### Mass spectrometry data analysis

The generated raw mass spectrometry files were analyzed with MaxQuant software [43] (version 1.3.0.5, using Andromeda search engine) against UniProtKB *D. melanogaster* database containing 18,826 entries (downloaded in April 2013) supplemented with common contaminants and concatenated with the reverse sequences of all entries. The following Andromeda search parameters were set: carbamidomethylation of cysteines as a fixed modification, oxidation of methionine and N-terminal acetylation as a variable modification, and Lys-C specificity with no proline restriction and up to two missed cleavages. The MS survey scan mass tolerance was 7 ppm and for MS/MS 20 ppm. For protein identification, minimum of 5 amino acids per identified peptide and at least 1 peptide per protein group were required. The false discovery rate was set to 1% at both peptide and protein levels. “Re-quantify” was enabled, and “keep low scoring versions of identified peptides” was disabled. The mass spectrometry analysis detected 1506 proteins in total (Additional file 1: Data S1). Average fold change and standard deviation between the control (*Mhc-Gal4/OregonR)* and the experiment (GFP-tagged muscle-driven Dg, *Mhc-Gal4/UAS Dg::GFP)* are determined from 3 independent biological replicates. Two-tailed Student’s *t* test was applied for statistical analysis. The numbers in the table correspond to the absolute numbers of reads per protein. Proteins with statistically significant ≥ 2-fold increase in comparison with control were considered as Dg-interacting proteins. Such analysis allowed identifying 83 muscle-specific Dg-interacting components (Additional file 1: Data S1). Note that some of the proteins, such as Zipper (myosin heavy chain) and WupA (troponin), were detected as separate isoforms (outlined by grayish in the Additional file 1: Data S1), Dg per se is marked by blue.

### Bioinformatical analyses

To arrange identified interactors in the functional protein-association network, we used STRING v10 database [44], with medium confidence score (0.04), and prediction methods that included neighborhood, gene fusion, co-occurrence, co-expression, experiments, databases, and text mining. The network is presented in a “confidence” view, where the thickness of lines connecting the nodes represents the confidence of association. Lines connect components clustered by MCL (Markov clustering algorithm) into protein complexes. Dashed lines show associations between components that do not form protein complexes. For Gene Ontology enrichment analysis, STRING and Gene Ontology Consortium [45] (http://geneontology.org) were used. To assign protein cellular localization and molecular function and to find human orthologs, the FB2016_02 release FlyBase was applied. To search for the human disease association, http://www.flyrnai.org [46] and http://www.genecards.org were used. For human disease association enrichment analysis, the entry of 77 human genes (orthologs of identified Dg-interacting components) was examined with http://ctdbase.org/tools (Disease) tool. For protein domain structure analysis, SMART (http://smart.embl-heidelberg.de) was used. For WW domain sequence alignment, PRALINE multiple sequence alignment source (http://www.ibi.vu.nl) was utilized.

### Histology and immunohistochemistry of *Drosophila* muscles

For the analysis of adult indirect flight muscle (IFM) morphology, 7-μm paraffin-embedded sections were cut from fly thoraces. To prepare *Drosophila* muscle sections, the fly bodies were immobilized in the collars in the required orientation and fixed in Carnoy fixative solution (6:3:1 ethanol to chloroform to acetic acid) at 4 °C overnight. Tissue dehydration and embedding in paraffin were performed as described previously [47]. Histological sections were prepared using a Hyrax M25 (Zeiss) microtome and stained with hematoxylin and eosin as described previously [13]. All chemicals for these procedures were obtained from Sigma Aldrich.

Larval body wall muscles were prepared as previously described [48]. Adult ovaries and guts were rapidly dissected in PBS and fixed in 4% formaldehyde (Polysciences, Inc.) for 15 min. Staining was performed as described [49]. Samples were mounted in 70% glycerol.

#### Antibodies for *Drosophila* immunohistochemistry

The following are the antibodies for *Drosophila* immunohistochemistry: polyclonal rabbit anti-Dg 1:1000 [50], anti-Kibra 1:1000 (gifts from Wu-Min Deng), and anti-gamma 1 (LanB2) 1:1000 (Abcam, ab47651); chicken anti-GFP 1:2000 (Invitrogen); monoclonal mouse anti-Hts (also called Adducin, 1:20, DSHB); and Alexa 488, 568, or 633 goat anti-mouse, anti-rabbit, anti-chicken (1:500, Molecular Probes). To visualize nucleoli, a 10-min-long incubation with 1× DAPI (Sigma Aldrich) in PBS was performed.

### Fractionation of mouse muscle tissue

All animal experiments were consistent with the Israel Council on Animal Experiments guidelines and the Institutional Regulations of Animal Care and Use. Specialized personnel provided mouse care in the institutional animal facility. To obtain whole-cell extracts, mouse tibialis anterior muscles (50 mg each) were homogenized as previously described [51]. Briefly, following homogenization in lysis buffer (20 mM Tris pH 7.2, 5 mM EGTA, 100 mM KCl, 1% Triton X-100, 1 mM PMSF, 3 mM benzamidine, 10 μg/ml Leupeptin, 50 mM NaF, and 2.7 mM Sodium OrthoVanadate), the homogenates were centrifuged at 6000 × *g* for 20 min at 4 °C, and the supernatant (containing whole-cell extract) was collected and stored at − 80 °C.

### Immunoprecipitation from mouse muscle extracts

For β-dystroglycan immunoprecipitation, a specific antibody was added to whole-cell extracts (control sample contained mouse IgG), samples were incubated overnight at 4 °C, and protein A/G agarose was then added for 4 h. To remove nonspecific or weakly associated proteins, the resulting precipitates were washed extensively with ten bed volumes of each of the following buffers: buffer A (50 mM Tris-HCl pH 8, 500 mM NaCl, 0.1% SDS, 0.1% Triton, 5 mM EDTA), buffer B (50 mM Tris-HCl pH 8, 150 mM NaCl, 0.1% SDS, 0.1% Triton, 5 mM EDTA), and buffer C (50 mM Tris-HCl pH 8, 0.1% Triton, 5 mM EDTA). Protein precipitates were eluted in protein loading buffer containing 50 mM DTT and were analyzed by SDS-PAGE and immunoblotting-specific antibodies as described [51].

#### Antibodies

The β-dystroglycan antibody (used for immunoprecipitation from mouse muscle extracts) developed by Glenn E. Morris (RJAH Orthopaedic Hospital, Oswestry, UK) was obtained from the Developmental Studies Hybridoma Bank, created by the NICHD of the NIH and maintained at The University of Iowa, Department of Biology, Iowa City, IA 52242 (MANDAG2, clone 7D11, lot# S1ea). The Kibra antibody was from Abcam (ab216508).

### Mouse myotubes differentiation

For immunofluorescence studies, mouse C2C12 myoblasts (ATCC) were seeded on poly-l-lysine-coated glass coverslips and cultured for 2 days in growth medium (GM), consisting of complete Dulbecco’s modified Eagle’s medium (DMEM) (Invitrogen) supplemented with 10% fetal bovine serum. At confluency, GM was replaced by differentiation medium (DM, DMEM plus 5% horse serum) and cells were grown for additional 5 days in DM. Cells were washed, fixed, and stained as described previously [52].

### Mouse muscle tissue sections

Mouse muscles were rapidly dissected, placed in disposable vinyl specimen molds (Tissue-Tek Cryomold), filled with O.C.T. Compound (Tissue-Tek), and frozen in − 60 °C cooler, as previously described [47]. Then, 10-μm-thick muscle sections were made on Microm HM 560 Cryostat (Thermo Scientific) and preserved at − 20 °C freezer till immunostaining procedure.

#### Antibodies for mouse immunohistochemistry

To detect Dg, the following antibody was used: goat anti-DAG1 (1:100, Abcam, ab136665). To detect Kibra, the following antibody was used: rabbit anti-Kibra (1:100, Abcam, ab216508). Donkey anti-goat Alexa 488 (1:500, Invitrogen, A32814) and goat anti-rabbit Alexa 568 (1:500, Invitrogen, A11011) were used as secondary antibodies.

### Microscopy and image analyses

Images of larval body wall muscles and ovarian and digestive system muscles were obtained using Zeiss LSM700 confocal laser scanning microscope. Protein expression patterns and protein co-localizations were analyzed from confocal images taken in a *z*-stacks (1-μm step). Images were processed with ZEN 2011 and Adobe Photoshop software.

The analysis of adult muscle sections was performed using the light microscope Zeiss Axiophot, the Zeiss AxioCam HRc camera, and the Zen 2011 blue software. The frequency of muscle degeneration was quantified as a ratio of degenerated muscles to the total number of analyzed muscles per genotype. The analyzed IFM sections were located at a position 200–250 μm to the posterior of the fly thorax.

Cross-sectional areas of all 12 indirect flight muscles were measured using Metamorph software (example areas are marked and outlined in the main figure) from images taken with Zen 2011 blue software. The edges of myofibers were estimated based on the hematoxylin and eosin staining.

All muscle analyses were performed from three biological replicates. Two-tailed Student’s *t* test was applied for statistics.

## Results

### Mass spectrometric profiling of the dystroglycan complexome in adult muscles

Currently, only a subset of diagnosed cases characterized by similar muscular dystrophy symptoms can be linked to mutations in known DGC components. This implies that additional, unknown Dg regulators are involved in muscular dystrophy development. To reveal new Dg-interacting partners that form complexes with Dg specifically in the muscle tissue, we took advantage of *Drosophila* as a recognized model to study muscular disorders and an exemplary system for molecular genetic manipulations [13, 18, 20, 21, 53, 54]. We expressed the C-terminal GFP-tagged version of full-length Dg [17] with the muscle-specific promoter of the *myosin heavy chain* (*Mhc*) gene and immune-isolated protein complexes from whole lysates of young (7-day-old) adult animals with further detection of specific Dg-interacting partners by mass spectrometry (Fig. 1b). To decrease false-positive detections and obtain reliable data, the experiments were performed in biological triplicates, and each experiment included its own control (Fig. 1c, Additional file 1: Data S1). As a result of mass spectrometric profiling of the Dg complexome from adult muscle tissue, we detected Dg protein itself plus 83 proteins that potentially can be directly or indirectly (via other partners) associated with Dg (Additional file 2: Table S1, Additional file 1: Data S1). Next, we performed intensive bioinformatics analyses on the identified proteins, assigned their subcellular localization and molecular function (Fig. 1d, e; Additional file 2: Table S1), and identified significant enrichment in various signaling pathways (Additional file 2: Table S2). Among the 83 identified Dg-associated proteins (Fig. 1e), bioinformatical analysis reveals 5 ECM components, 6 proteins associated with the plasma membrane, 16 cytoskeleton structural constituents, 8 mitochondria-linked factors, 9 nuclear, 21 cytosolic, 5 endoplasmic reticulum-associated, and 2 cytoplasmic vesicle-linked proteins (the numbers of identified proteins that are associated with different subcellular compartments are shown in red squares, see also Additional file 2: Table S1). Molecular function and subcellular localization of 20 components remain unknown. Particularly, in the Dg interactome, we identified a significant enrichment in 2 metabolic pathways: the glutathione metabolism controlling pathway (*p* = 0.021) and the oxidative phosphorylation pathway (*p* = 0.036; Additional file 2: Table S2). Previously, we reported that Dg loss-of-function mutants have impaired metabolism [39], and their muscles are particularly sensitive to sugar dietary restriction [18]. Additionally, among the immune-isolated proteins upon the GFP-tagged Dg overexpression, we found a key enzyme that regulates the glycerol metabolic process to guard flight behavior, glycerophosphate oxidase 1 (Gpo-1) [55].

Moreover, we identified the closest human homolog for each Dg-interacting partner, searched for their association with diseases and pathological condition(s) in humans, and distinguished their significant enrichment in disease associations (Additional file 2: Tables S1 and S3). Importantly, most of the identified Dg-associated components are evolutionarily conserved and have human homologs (Additional file 2: Table S1), the majority of which are linked to muscular, neuronal, or metabolic disorders. Interestingly, the human disease association enrichment analysis (Additional file 2: Table S3) identified that human homologs of Dg-interacting proteins detected in this study have significantly enriched associations with cardiovascular, digestive system, and urogenital diseases. Taking into account that MD patients often have cardiac dysfunctions [56], markedly disturbed gastrointestinal motor function [57], and urinary incontinence [58], we are optimistic that further research on these Dg-associated components will help in revealing the etiology of these pathologies.

In summary, among the identified Dg-associated proteins, there are components of the basal lamina, proteins that play roles in transmembrane transport, cytoskeletal proteins, lectins, components involved in protein biosynthesis and degradation, factors that control cellular metabolism, endoplasmic reticulum and Golgi apparatus constituents, mitochondria-associated proteins, components of the nucleosome, and Hippo signaling factors (Fig. 1d, e).

### Dg binds extracellular LamininA and cytoskeletal proteins in *Drosophila* muscles

**Fig. 2:**
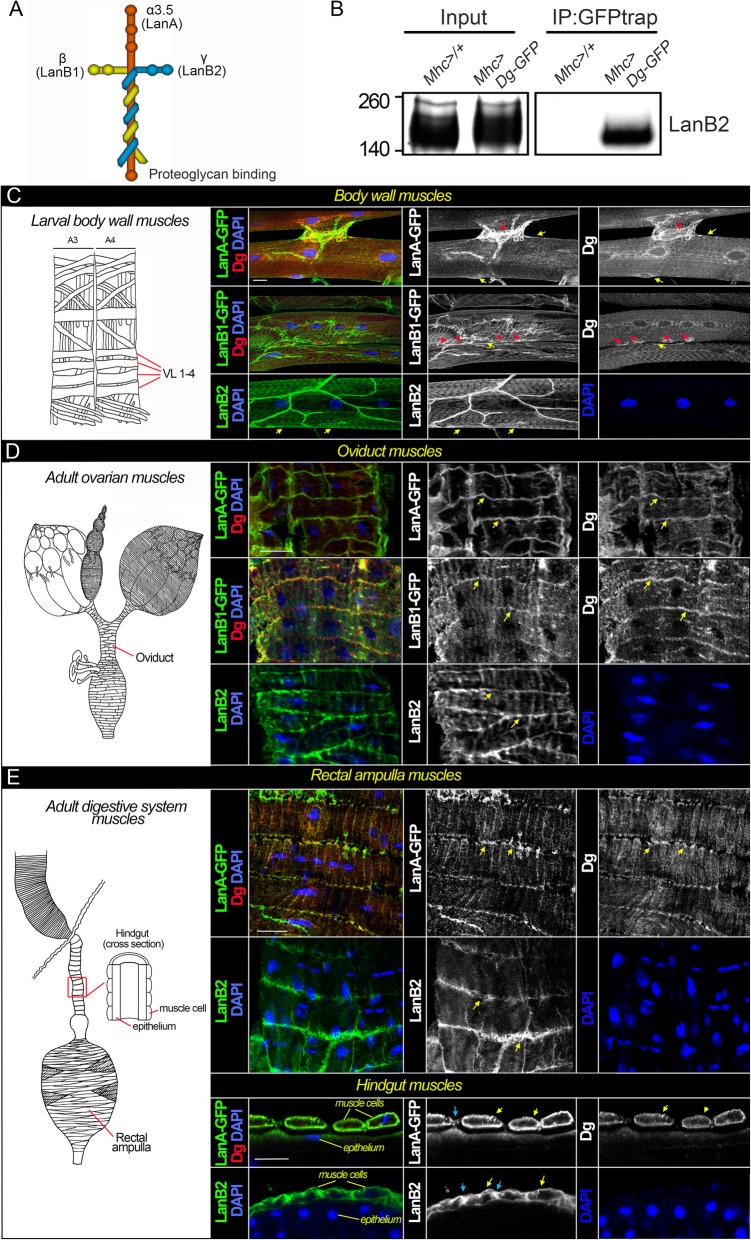
Dg associates with three laminin subunits in the muscles. **a** Schematic drawing of the LamininA heterotrimer containing α3.5 (encoded by LamininA, LanA gene), β (encoded by LamininB1, LanB1), and γ (encoded by LamininB2, LanB2) chains. **b** Co-IP verifies Dg and LanB2 interaction in the muscles. **c**–**e** Dg and laminin protein co-localization in the larval body wall (**c**), adult oviduct (**d**), and adult rectal ampulla and hindgut (**e**) muscles. **c**–**e** (Left panels) schematics of the larval body wall muscles, adult ovarian muscles, and adult digestive system muscles (drawings adapted from [18]). The striated larval body wall muscles are the functional equivalents of vertebrate skeletal muscles. Although striated, the visceral muscles surrounding the gut and gonads are functionally analogous to vertebrate smooth muscles. **c** (Right panel) confocal images of the larval body wall muscles (VL1-4) presented as maximum intensity projections from multiple *z*-sections. Yellow arrows show Dg and laminin subunit co-localization at the sarcolemma and red arrows point to the Dg- and Lan-enriched NMJs. **d** (Right panel) images of single *z*-sections of the oviduct muscles. Yellow arrows show Dg and laminin co-localization at the muscle cell membrane. **e** (Right panel) images of single *z*-sections of the rectal ampulla muscles and cross-sections of hindgut muscle cells (for orientation, see the scheme). Yellow arrows show Dg and Lan co-localization at the muscle cell membrane, and blue arrows point to the muscle attachment sites. Dg—red; LanA, LanB1, and LanB2—green; DAPI—blue. Expression patterns for Dg, laminins, and DAPI are also shown in separate channels

Next, we wanted to verify at the tissue level, using western blotting and immunohistochemistry assays, Dg interaction and co-localization with the proteins identified by the proteomics approach. Muscles are coated by a layer of ECM material called the basement membrane (BM). BM is not only a stationary structure that offers mechanical support and physical barrier for the muscle cell, it also provides a scaffold to orient cells during regeneration, plays an active role in myogenesis and synaptogenesis, and is lately viewed in the context of ECM intracellular signaling mediated by the membrane-associated receptors, including Dg. The composition of BMs is variable; however, it is usually comprised 2 meshwork-forming components, laminins and collagen. Laminins are a family of heterotrimeric glycoproteins composed of 3 different chains, α, β, and γ. The long arm of the laminin is tethered to an ECM receptor, such as Dg and/or integrins [59]. Mammals contain at least 15 laminin heterotrimers, which are formed through various combinations of 5α, 4β, and 3γ subunits, while invertebrates possess only 1–2 laminin heterotrimers. Four laminin chains are encoded by the *Drosophila* genome: α1.2 (encoded by the *wing blister*, *wb* gene), α3.5 (encoded by *LamininA*, *LanA* gene), β (encoded by *LamininB1*, *LanB1*), and γ (encoded by *LamininB2*, *LanB2*), forming 2 trimers—LamininA (α3.5, β, γ) and LamininW (α1.2, β, γ) [60, 61]. Our mass spectrometry detection identified all 3 subunits of LamininA to be co-purified with Dg from *Drosophila* muscles (Fig. 2a). Since the Dg-Laminin association was not previously tested in *Drosophila* muscles, we co-immunoprecipitated Dg::GFP and confirmed the Dg-Laminin association by western blotting assay. These data show that the muscle-expressed Dg forms a complex with laminin, as detected with anti-LanB2 antibodies (Fig. 2b).

To further evaluate the Dg-LamininA association at the cellular level, we performed immunohistochemical co-staining of these two proteins in muscle tissue. Dg protein has been previously shown to be expressed in multiple types of *Drosophila* muscles and mainly localized to the muscle sarcolemma [13, 18, 62]. We examined Dg-LamininA co-localization in the skeletal-like body wall muscles of *Drosophila* wandering larvae (Fig. 2c) and smooth-like visceral muscles from adult reproductive (Fig. 2d) and digestive (Fig. 2e) systems. We detected two Laminin chains, LanA and LanB1, by expressing GFP-tagged versions of these proteins, while LanB2 was detected with antibodies. We observed that Dg and LamininA subunits co-localize at the sarcolemma of the larval body wall, oviduct, rectal ampulla, and hindgut muscles (Fig. 2c–e), as well as at the larval neuromuscular junctions (NMJs) (Fig. 2c, red arrows) and at the sites of muscle attachments to the hindgut epithelium (Fig. 2e, blue arrows). These results suggest that similarly to mammalian Dg, *Drosophila* Dg binds LamininA in muscle cells.

In addition, we tested several other components of the muscle cytoskeleton for their association with Dg (Additional file 2: Figure S1). We used lines expressing GFP-tagged proteins [37] to visualize muscle expression patterns of Hts and WupA cytoskeletal components. Hts/Add (Hu li tai shao/Adducin) is an adducin homolog associated with the plasma membrane cytoskeleton that has an essential function in adult somatic muscle development [63]. Hts/Add is also deregulated at the NMJs in amyotrophic lateral sclerosis (ALS) patients and ALS-animal disease models [64]. Hts/Add is expressed in both muscles and neurons and is enriched at the pre- and post-synapse at the NMJs, where it controls synapse elaboration and elimination [65]. Hts/Add is associated with Na^+^/K^+^ ATPase, the ATP subunit ⍺, which was also co-immunoprecipitated with Dg in this study (Fig. 1e, Additional file 2: Table S1) and Coracle (Cora), which has been previously shown to be deregulated at the NMJs of *Dg* mutants [20]. At *Drosophila* NMJs, Dg is expressed at the post-synapse, and its absence influences the localization of proteins involved in neurotransmission, which abolishes NMJ functions [17, 20]. Now we identified that Dg and Hts/Add are associated in the *Drosophila* muscle tissue by testing Dg and Hts/Add protein co-localization patterns. Importantly, we confirmed this association at the cellular level in different muscle types (Additional file 2: Figure S1B, D, E). We observed that in all muscle types, Hts/Add is present in puncta at the sarcolemma where it co-localizes with Dg (Additional file 2: Figure S1B, D, E). Moreover, in the larval body wall muscles, strong enrichment of both proteins was observed at the NMJs (Additional file 2: Figure S1B). In addition, by western blotting assay, we confirmed that Hts/Add protein forms a complex with Dg in adult muscles (Additional file 2: Figure S1C).

Next, we addressed Dg’s association with muscle thin filament protein, Wings up A (WupA), which is the component of the troponin complex involved in Ca^2+^-dependent regulation of muscle contraction. WupA also has been shown to be involved in the regulation of heart, skeletal, and flight muscle development [66, 67]. Now, our expression data show that in the muscles, Dg co-localizes with the troponin complex component (Additional file 2: Figure S1), suggesting that Dg could be involved in the regulation of muscle contraction. In support of this, we also have found several additional proteins involved in myosin motor-based movement along actin filaments to be associated with Dg, such as tropomodulin, two troponin C isoforms, myosin light chain C, myosin alkali light chain, actin 42A, crinkled, gelsonin, and spaghetti squash (Fig. 1e, Additional file 2: Table S1).

In summary, by analyzing Dg protein co-localization and co-immunoprecipitation, we validated that proteins identified by mass spectrometry, such as LanA, LanB1, LanB2, Hts/Add, and WupA, indeed associate with Dg in muscle tissue. It implies that the candidates identified in our screen can be used as a basis for further investigation of Dg interacting partners, manipulation of which could combat the progression of muscle dystrophies and cachexia.

### Dg and the Hippo kinase signaling pathway interact to sustain muscle integrity

**Fig. 3:**
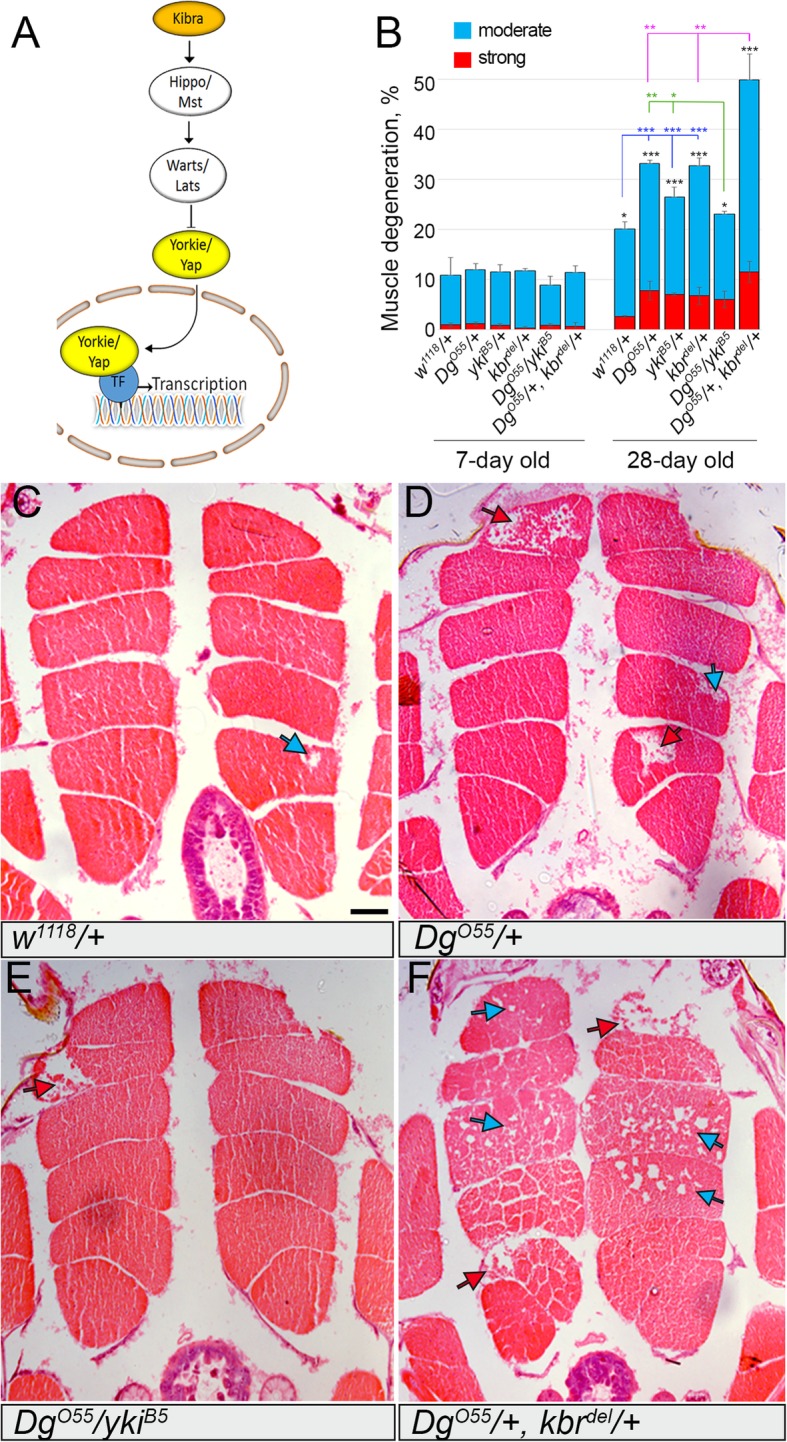
Hippo signaling pathway components modify the Dg-linked muscle degeneration phenotype. **a** Scheme of the Hippo kinase signal transduction pathway, major components of which are evolutionarily conserved from flies to humans. Kbr is a Hippo signaling activator. Wts/Lats phosphorylates transcriptional co-activator Yki, which prevents its relocation from the cytosol to the nucleus. Two components of the pathway Kbr (orange) and Yki (yellow) were identified by the mass spectrometry analysis to be associated with the Dg protein. **b** Bar graph represents the quantification of the moderate and strong IFM degeneration phenotypes (blue and red bars, respectively) in young (7-day-old) and aged (28-day-old) animals (Additional file 2: Table S5). Aging per se causes significant muscle degeneration (see 28-day-old control *w*^*1118*^*/+*, and black stars indicate statistical significance. Reduction of *Dg*, *kbr*, or *yki* by one copy additionally increases moderate—and in particular, strong—muscle degeneration in old animals (see 28-day-old *Dg*^*O55*^*/+*, *yki*^*B5*^*/+*, *kbr*^*del*^*/+* and blue stars for statistics). The introduction of the *Hippo* pathway mutations into a *Dg* heterozygous background significantly modifies the age-dependent muscle degeneration phenotype (Additional file 2: Table S5). In particular, the reduction of *yki* by one copy suppresses (see *Dg*^*O55*^*/yki*^*B5*^ and green stars for statistics), while reduction of Kbr by one copy enhances (see *Dg*^*O55*^*/+*, *kbr*^*del*^*/+*, and pink stars for statistics) the frequency of muscle degeneration observed in *Dg* heterozygous mutants. **c**–**f** Hematoxylin and eosin (H&E)-stained transverse sections of indirect flight muscles (IFMs) of 28-day-old control (*w*^*1118*^*/+*), *Dg* heterozygous (*Dg*^*O55*^*/+*), *Dg* and *yki* trans-heterozygous (*Dg*^*O55*^*/yki*^*B5*^), and *Dg* and *kbr* trans-heterozygous (*Dg*^*O55*^*/+*, *kbr*^*del*^*/+*) animals. Blue and red arrows point to moderate and strong muscle degeneration, respectively

Interestingly, together with Dg, we co-purified Kbr and Yki (Fig. 1e, Additional file 2: Table S1), which are the two members of the key evolutionarily conserved mechanotransducing Hippo signaling pathway [27, 68]. Since mechanical signals regulate many aspects of muscle cell behavior, including growth, differentiation, and motility, we wanted to analyze in greater detail whether Dg acts in concert with Hippo signaling to regulate muscle growth and maintenance. The Hippo signaling cascade was first discovered and characterized in *Drosophila* (reviewed in [69, 70]). To transduce signals, Kbr together with Merlin (mammalian neurofibromatosis type II, NF2) recruits the scaffolding protein Salvador (mammalian WW45), which forms a complex with the Hippo kinase (Hpo) or its mammalian homolog Mst to phosphorylate and activate the DBF family kinase Warts (mammalian Lats, Fig. 3a). Activated Warts in association with the adaptor protein Mats (mammalian Mob) phosphorylates and inhibits nuclear translocation of another Dg-interacting protein found in this study, the non-DNA-binding cofactor Yki (mammalian Yap or Taz, Fig. 3a). Yki protein is a downstream effector of the Hippo signaling cascade directly involved in the activation of gene expression, while Kbr and Hpo are upstream components involved in the cascade activation resulting in Yki’s phosphorylation and cytoplasmic retention, thus downregulation of Yki activity. Therefore, Kbr-mediated pathway activation results in the inactivation of the transcriptional regulator Yki (Fig. 3a). Intriguingly, our data show that Dg can associate and form complex with both Kbr and Yki, which have opposite roles in the Hippo signaling cascade. Since the role of Dg in this signaling pathway is not fully understood, we further tested these interactions. Particularly, we addressed whether Dg and the Hippo kinase signaling pathway are physically and functionally interconnected and whether this association is biologically relevant for muscle function and MD development using several approaches.

Firstly, we evaluated Yki and Kbr expression in *Drosophila* muscles (Additional file 2: Figure S2 and S3). We detected Kbr expression by antibody staining in the cytoplasmic puncta in different types of muscles (Additional file 2: Figure S2A-C). For Yki detection, we analyzed the muscles of animals expressing GFP-tagged Yki [37] and identified patterns similar to Kbr expression (Additional file 2: Figure S3A-C). Moreover, both Kbr and Yki co-localized with Dg at the puncta adjacent to the muscle cell membrane (Additional file 2: Figure S2D and S3D).

Secondly, we tested the biochemical association between Dg and Hippo signaling. Intriguingly, the association of Dg and Dys in human and *Drosophila* relies on WW domain-mediated protein interactions [13, 19, 71, 72]. In fact, the cytoplasmic region of Dg protein contains two functional WW domain-binding PPxY motifs, whose function can be regulated by tyrosine phosphorylation, which affects Dg-Dys interaction [72]. Similar to Dys, *Drosophila* Kbr protein, identified by mass spectrometry to be associated with Dg, also contains two WW domains (Additional file 2: Figure S4). Furthermore, it has been shown that the core components of the *Drosophila* Hippo pathway, like in mammals, associate with each other via PPxY motif-WW domain interactions [73]. We specifically focused on Dg interaction with a critical component of this mechanotransducing pathway, Kbr. While binding of Dg peptide to Kbr WW tandem has been recently reported [74], Dg-Kbr interaction has not been demonstrated in any animal in vivo*.* We expressed a GFP-tagged Dg protein using *Mhc-Gal4* and immunoprecipitated complexes from whole fly extracts using anti-GFP beads. Immunoblotting with anti-GFP antibodies allows recognition of the *Dg::GFP* fusion protein only in extracts from *Mhc>Dg::GFP*. Importantly, immunoblotting with an anti-Kbr antibody suggests that Kbr and Dg can associate and form complex (Additional file 2: Figure S2E). To assess the possibility of non-specific interactions between Kbr and GFP, we also overexpressed membrane-tagged GFP using the same driver (*Mhc>CD8::GFP*). Immunoblotting with anti-Kbr confirms that the interaction with Kbr is specific for Dg (Additional file 2: Figure S2F). We propose that this interaction may occur via PPxY motif-WW domain interactions; however, this hypothesis needs to be further tested by mutagenesis of the putatively interacting domains. In addition, *Drosophila* Yki also contains WW domains (Additional file 2: Figure S4), and Dg-Yap binding through PPxY-WW interaction has been recently demonstrated in mice [36]. However, a recent study showed that in human cells, Dg can bind the WW tandem domain of Kbr, but not Yki/Yap [74]. Therefore, further studies are required to further dissect the biochemistry of Dg interaction with these Hippo signaling factors.

Thirdly, we wanted to test whether Dg functionally interacts with the Hippo pathway components and whether this interaction is important for the maintenance of adult muscle tissue. A great tool to test for a functional interaction between genes is the genetic interaction approach. It evaluates the effect of pairwise gene deletion or gene expression inhibition on the outcome of a particular phenotype. On one hand, this analysis allows to identify the functional association between genes, even if their physical interaction is weak or transient. On the other hand, genetic interaction screens cannot discriminate between direct and indirect interactions; however, they provide evidence for involvement in the same pathway or process and give additional information about gene function and pathways. To test for the functional interaction of Yki and Kbr with Dg, we generated trans-heterozygous mutants that had reduced by one copy Dg and Kbr or Yki and analyzed the integrity of their muscles.

In particular, we analyzed the morphology of indirect flight muscles (IFMs) in young (7-day-old) and aged (28-day-old) animals and quantified the muscle degeneration phenotype (Fig. 3b, Additional file 2: Table S4). IFMs are fast-twitching muscles with high mitochondrial content necessary for long-lasting oxidative metabolism. In addition to calcium stimulation, these asynchronous muscles require stretch activation to induce their contractions [75]. In young animals, no significant increase in muscle degeneration was observed in animals bearing only one copy of *Dg*, *yki* or *kbr* (*Dg*^*O55*^*/+*, *yki*^*B5*^*/+*, or *kbr*^*del*^*/+*, respectively) or in flies trans-heterozygous for *Dg* and Hippo pathway components (*Dg*^*O55*^*/yki*^*B5*^ or *Dg*^*O55*^*/+*; *kbr*^*del*^*/+*), when compared to controls (*w*^*1118*^*/+*, 7 days old, Fig. 3c–f). Aged control flies had a substantial muscle degeneration phenotype (*w*^*1118*^*/+*, 28 days old); moreover, heterozygous *Dg*^*O55*^*/+*, *yki*^*B5*^*/+*, or *kbr*^*del*^*/+* animals displayed significantly increased muscle degeneration (28 days old, Fig. 3b), suggesting that the reduction of the Hippo signaling components by one copy, similarly to reduction of Dg, leads to age-dependent muscle degeneration. Interestingly, this phenotype was characterized by either moderate or strong changes in the muscle morphology resulting in muscle fiber loss (blue and red arrows and bars, respectively, Fig. 3c–f).

Importantly, in the muscle tissue, the trans-heterozygous combination of *Dg* and *kbr* (*Dg*^*O55*^*/+*; *kbr*^*del*^*/+*) resulted in a significant increase of both strong and moderate muscle degeneration phenotypes, when compared to respective controls (28 days old, Fig. 3b). At the same time, the reduction of *yki* expression by one copy in the *Dg* heterozygous background partially rescued the age-dependent muscle degeneration phenotype. These data show that in adult muscles, Dg genetically interacts and cooperates with Kbr to promote the Hippo signaling cascade required to maintain muscle tissue integrity during aging and suggest that Dg acts to repress the nuclear function of Yki.

In summary, our results demonstrate that in the muscles, the transmembrane protein Dg physically and functionally interacts with the Hippo signaling pathway, in particular, the WW domain proteins, Yki and Kbr, and this interaction is required to sustain muscle integrity during aging.

### The Hippo kinase signaling pathway cell autonomously regulates adult muscle integrity

Currently, Hippo signaling has not been shown to play a role in adult muscle maintenance in *Drosophila.* Since we identified that during aging, Dg and the Hippo signaling components interact to maintain muscle integrity, we wanted to analyze the Hippo pathway requirement in adult muscles. Similar to Dg, the components of the Hippo pathway are expressed in various tissues; therefore, we wanted to test whether Hippo signaling is involved in the cell-autonomous regulation of muscle integrity. In particular, we analyzed the consequences of the muscle-specific down- or upregulation of Hippo pathway components using different *Gal4* drivers. Importantly, the downregulation of Yki with *Mef2-Gal4* driver resulted in the complete loss of indirect flight muscles, IFMs (Additional file 2: Figure S5), while its downregulation with *Mhc-Gal4* resulted in the appearance of flies with relatively normally developed muscles. Mef2 (myocyte enhancer factor 2) is a transcription factor required for the activation of a large number of muscle protein genes essential for muscle development, while Mhc (myosin heavy chain) is the motor protein providing the force for muscle contraction and is expressed in already differentiated muscles. Based on these observations, we conclude that Yki is essential for muscle development and that the *Mhc-Gal4* driver is more suitable to assay the role of Yki and other Hippo pathway mutants in muscle maintenance during adulthood.

**Fig. 4:**
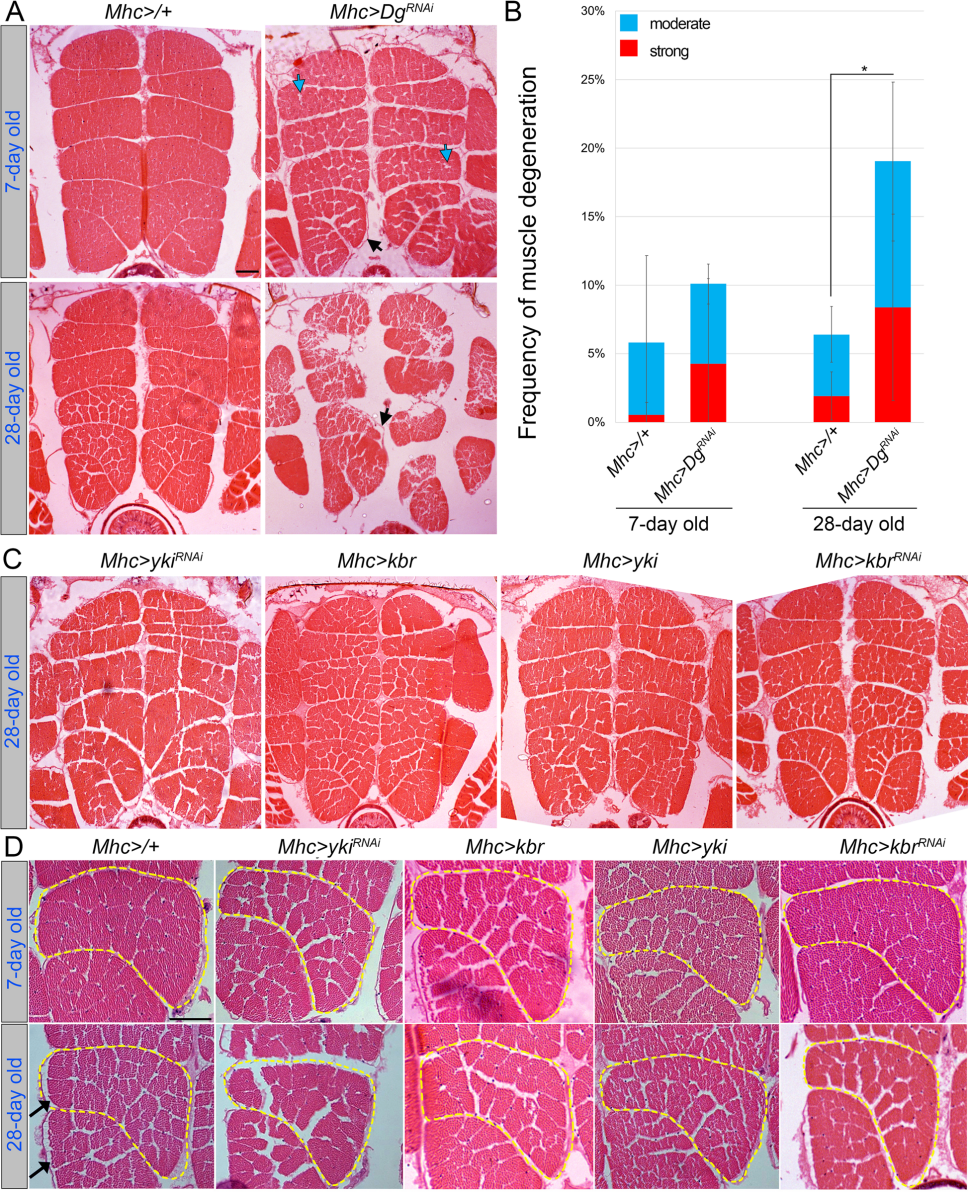
Hippo kinase signaling pathway controls muscle size. **a** Images of transverse sections of IFMs from young and aged (7- and 28-day-old) controls (*Mhc-Gal4/+*) and mutant flies with the muscle-specific *Dg* downregulation (*Mhc>Dg*^*RNAi*^). Note the appearance of moderate muscle deterioration upon Dg downregulation in young flies (blue arrows) and strong muscle deterioration in aged flies (black arrows). **b** The bar graph shows the frequencies of moderate (blue bars) and strong (red bars) muscle degeneration in controls and in flies with *Dg* downregulation in the muscles. **c** Images of transverse sections of IFMs of aged mutant flies with *kbr* and *yki* up- or downregulation in the muscles. Note that in all genotypes, no obvious muscle degeneration is observed; however, the muscles appeared to be smaller in size. ***p ≤ 0.001; **p ≤ 0.01; *p ≤ 0.05. **d** Enlarged view of IFMs. Downregulation of *Dg* (*Mhc-Gal4>Dg*^*RNAi*^) and up- and downregulation of *yki* (*Mhc-Gal4 >yki*^*RNAi*^, *Mhc-Gal4>yki*) or *kbr* (*Mhc-Gal4>kbr*^*RNAi*^, *Mhc-Gal4>kbr*) result in the appearance of smaller muscles, when compared to control (*Mhc-Gal4/+*). The yellow dashed line outlines an individual muscle (muscle 5). Black arrows point to muscle atrophy, which is detected by smaller muscle cells clearly separated from the neighboring cells

As negative and positive controls, we used heterozygous *Mhc-Gal4/+* flies and flies that had *Dg* downregulated by *RNAi* in the muscles using the same driver (Fig. 4a)*.* The frequencies of degenerated muscles were similar in young control and mutant flies that had Dg downregulated either in the whole organism (*Dg*^*O55*^*/+*) or in the muscle tissue specifically (*Mhc-Gal4>Dg*^*RNAi*^). However, aged animals with a muscle-specific reduction of Dg showed lower percentages of degenerated muscles than Dg heterozygotes (compare Fig. 3b and 4b, Additional file 2: Table S4). Since it is known that Dg has additional important functions, for example, in the nervous system, these data suggest that during aging, Dg expression in non-muscle tissues also contributes to muscle maintenance.

Notably, when we analyzed aged animals with reduced Dg levels in the muscles, we observed that the appearance of muscle tissue was altered, which manifested as the apparently increased space between muscles (Fig. 4a, black arrows). In particular, muscles of aged flies separated from the epimysium, a fibrous tissue enveloping each muscle (Fig. 4a, black arrows). Such morphological changes do not necessarily cause myofiber loss or degeneration, but rather appear as a result of the decreased mass and size of myofibers and muscles. This phenotype, observed in *Drosophila*, resembles the clinically described muscle atrophy in humans, which is the reduction in muscle mass leading to muscle weakness. Importantly, similar defects were observed in young and aged Hippo signaling mutants (Fig. 4c, d). Notably, muscle atrophy is often observed in various diseases, including cancer, AIDS, congestive heart failure, chronic obstructive pulmonary disease, and renal failure. Moreover, starvation and age-related sarcopenia ultimately lead to muscle atrophy. Muscle atrophy can also develop to “cachexia” or wasting syndrome with poor prognosis and recently; Hippo signaling has been associated with cachexia in a mouse cancer model [76]. Therefore, we wanted to analyze the muscle atrophy phenotype caused by Dg and Hippo signaling defects in greater detail.

### Deregulation of Hippo signaling affects muscle size and results in age-dependent muscle atrophy

**Fig. 5:**
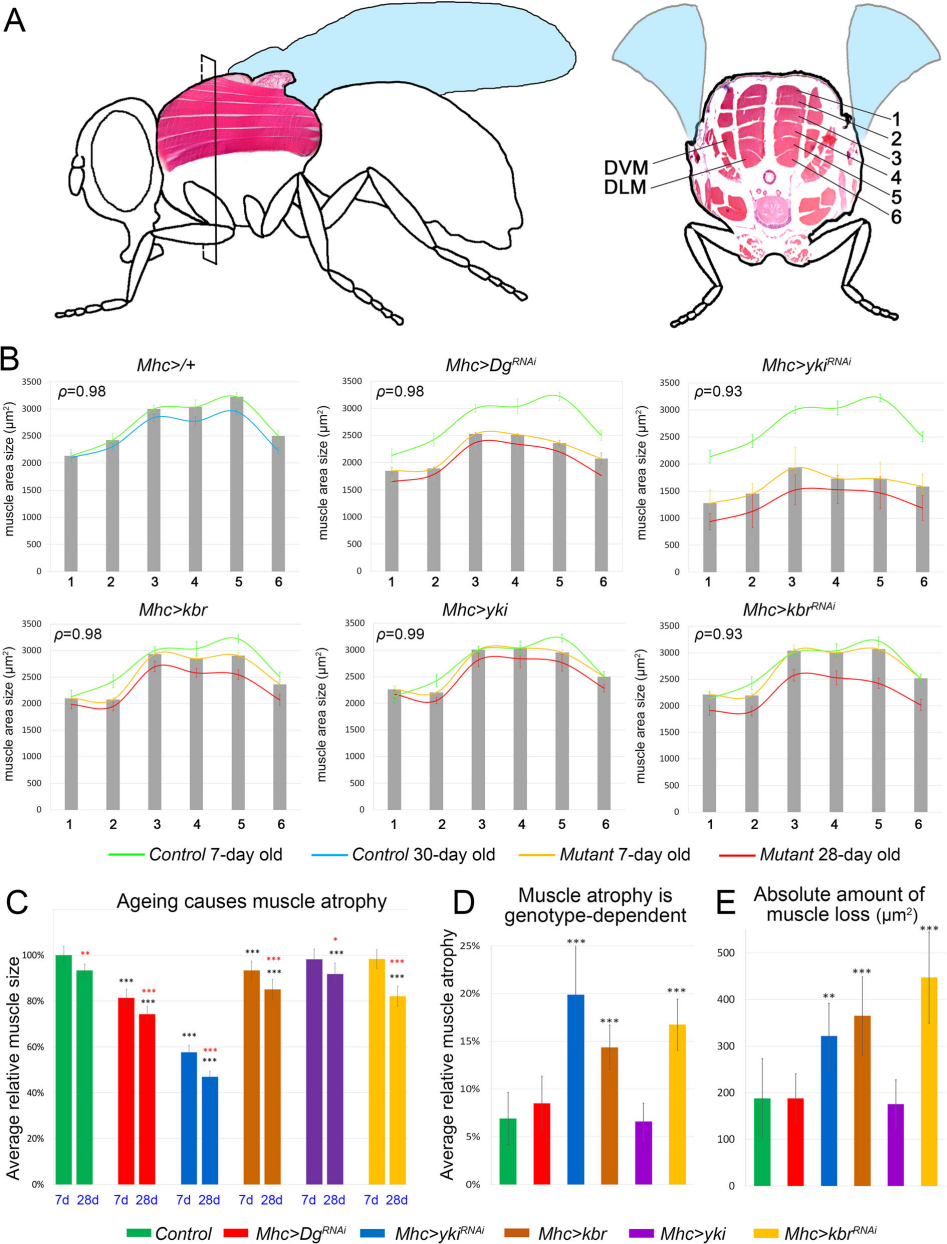
The Hippo pathway and Dg regulate muscle size and atrophy. **a** Schematic drawing of a fly from lateral and cross-section views indicates the position of the analyzed IFMs. The numbers for the pairs of dorsolateral muscles (DLM) are indicated in the cross-section view. **b** The bar graphs represent the average sizes of the muscles in different DLM pairs in young flies (7 days old) in controls or upon downregulation of *Dg* (*Mhc-Gal4>Dg*^*RNAi*^) and up- and downregulation of *yki* (*Mhc-Gal4>yki*^*RNAi*^, *Mhc-Gal4>yki*) and *kbr* (*Mhc-Gal4>kbr*^*RNAi*^, *Mhc-Gal4>kbr*). The green line outlines the averaged sizes of the young control muscles (shown in all genotypes for comparison), while the blue line indicates the aged control muscles. The yellow line corresponds to the averages of muscle size in mutant young animals, and the red line shows the average for aged animals of the corresponding genotype. Note that in all genotypes, on average, all muscles are similarly reduced in size. The correlation between the cross-sectional area of each muscle in young vs. old flies of the same genotype is shown at the top left corner of each graph (*ρ* = correlation index). Correlation is exclusively > 92%, signifying a nearly perfect positive correlation between 7 and 28 days (essentially, every muscle atrophies between day 7 and day 28). **c** The bar graph shows the average of relative muscle size in young and aged control and mutant flies. Aging causes a significant reduction in muscle size when compared to 7-day-old controls (black asterisks). Note that muscle size significantly decreases with age in all genotypes (red asterisks, compared to 7-day-old animals of the same genotype) (see also Additional file 2: Table S5). **d** The bar graph shows the amount of age-dependent muscle atrophy, when compared to 7-day-old animals of the same genotype (see also Additional file 2: Table S6). Note that muscle atrophy is differentially increased with age upon muscle-specific *yki* downregulation and *kbr* up- and downregulation. **e** The bar graph shows the absolute amount of age-dependent muscle atrophy (μm^2^). To measure this value, the averaged area of an individual muscle in old flies was subtracted from the corresponding value in young flies. Note that animals with *kbr* up- and downregulation have the highest amount of muscle size loss (see also Additional file 2: Table S6). ***p ≤ 0.001; **p ≤ 0.01; *p ≤ 0.05 (black, compared to 7-day-old control; red, compared to 7-day-old animals of the same genotype). Two-tailed Student’s *t* test was applied for statistical analysis

To evaluate the severity of muscle atrophy caused by muscle-specific Dg and Hippo signaling pathway deregulation, we analyzed the muscle size in control and mutant young and old animals. In particular, we focused on the indirect flight muscles (IFMs), as they represent one of the largest groups of adult muscles in *Drosophila* (Fig. 5a). IFMs do not directly drive wing movements but are involved in the deformation of the thorax to which the wing is attached, which indirectly leads to wing movements. These striated muscles closely resemble vertebrate striated muscles, as their organization, contraction, and relaxation are almost identical. These muscles are also extremely energy-dependent, since they are required for the exceptionally high wing beat frequency observed in insects [77]. These muscles contain a lot of mitochondria for long-lasting oxidative metabolism and little sarcoplasmic reticulum. There are 2 sets of IFMs—dorsal-ventral (DVM) and dorsal-longitudinal (DLM) muscles—which occupy a large part of the thoracic region and work antagonistically (Fig. 5a). Here, we measured the cross-sectional area of the DLMs. This group consists of 12 (6 on each side) muscles, elongated along the anterior-posterior axis and relatively similar in their cross-sectional appearance. This regularity is useful for quantitative and qualitative assessment of the muscle atrophy phenotype (Fig. 5a).

We found that in controls, the size of each DLM depends on its position, in that the most-dorsal and most-ventral muscles (pairs 1, 2, and 6) are smaller (~ 2200–2500 μm^2^), while the cross-sectional area of the longer muscles (pairs 3, 4, and 5) is larger (~ 3000 μm^2^, Fig. 5b, Additional file 2: Figure S6 and Table S6). Importantly, upon aging, the size of all muscles, regardless of their previous size or position, was significantly reduced in a similar manner (compare green and red lines for young and aged flies in Fig. 5b, Additional file 2: Figure S6). On average, in aged controls, the cross-sectional area of each muscle was ~ 10% smaller than in young flies (Fig. 5c, Additional file 2: Figure S6 and Table S6). This tendency for age-dependent muscle atrophy was observed for all genotypes. Importantly, the correlation analysis between 7- and 28-day-old muscles shows correlations exclusively > 92% (Fig. 5b, *ρ* = correlation index). Such significant positive correlation demonstrates that essentially every muscle has similar rates of atrophy upon aging.

In addition, young flies that had Dg and Yki levels reduced specifically in the muscles had dramatically smaller muscles (~ 20% and ~ 40% reduction of the muscle area, respectively), suggesting that Dg and Yki control proper muscle size already in young animals (Fig. 5c). Since all aged animals regardless of the genotype showed some degree of muscle atrophy upon aging, next, we measured the amount of muscle loss by comparing the average muscle area in aged vs. young animals of the same genotype (Fig. 5d). Since every muscle proportionally atrophied, we also assayed the absolute amount of muscle loss by subtracting the average muscle area of aged from the average muscle area of young flies (Fig. 5e). Interestingly, for mutants with Dg downregulation, the rate and the total amount of atrophy were not significantly different from controls, implying that while muscle-specific reduction of Dg levels during adulthood leads to age-dependent muscle degeneration (Fig. 4a, b), it does not accelerate age-dependent muscle atrophy (Fig. 5d, e; Additional file 2: Tables S5-S6). A similar tendency was observed in muscles with Yki upregulation, both the rate and the total amount of muscle loss were similar to controls. At the same time, Yki downregulation had a dramatic effect on age-dependent maintenance of muscle size. *Yki*^*RNAi*^ muscles were already significantly smaller in young animals; moreover, their size was additionally decreased upon aging (~ 20% and ~ 300 μm^2^, Fig. 5d, e; Additional file 2: Table S6)s. Similar results were obtained in mutants that had Kbr up- or downregulated. However, in comparison with Yki downregulation, the average relative muscle atrophy was lower, while the absolute amounts of muscle area loss were higher (~ 15% and ~ 400μm^2^, Fig. 5d, e; Additional file 2: Table S6).

Together, these data show that in *Drosophila*, the Hippo signaling pathway is involved in the control of age-dependent maintenance of muscle size. Importantly, Yki downregulation had a more dramatic effect on muscle growth already in young animals, suggesting that Hippo signaling activation has a negative effect on muscle growth. These data are consistent with the previous report that shows that in mammals, YAP/Yki positively regulates basal skeletal muscle mass and protein synthesis [32]. Also, we observed that the muscle-specific perturbation in Kbr expression (*Mhc>kbr*^*RNAi*^ and *Mhc>kbr*) significantly increases muscle atrophy upon aging, when compared to respective young mutants (Fig. 5c–e, Additional file 2: Tables S5-S6). Since age-dependent muscle atrophy was observed upon both downregulation and overexpression of Kbr, we conclude that during adulthood, the proper balance in Hippo signaling activity is important for muscle size maintenance.

### Dg-Kbr association is conserved in mammals

**Fig. 6:**
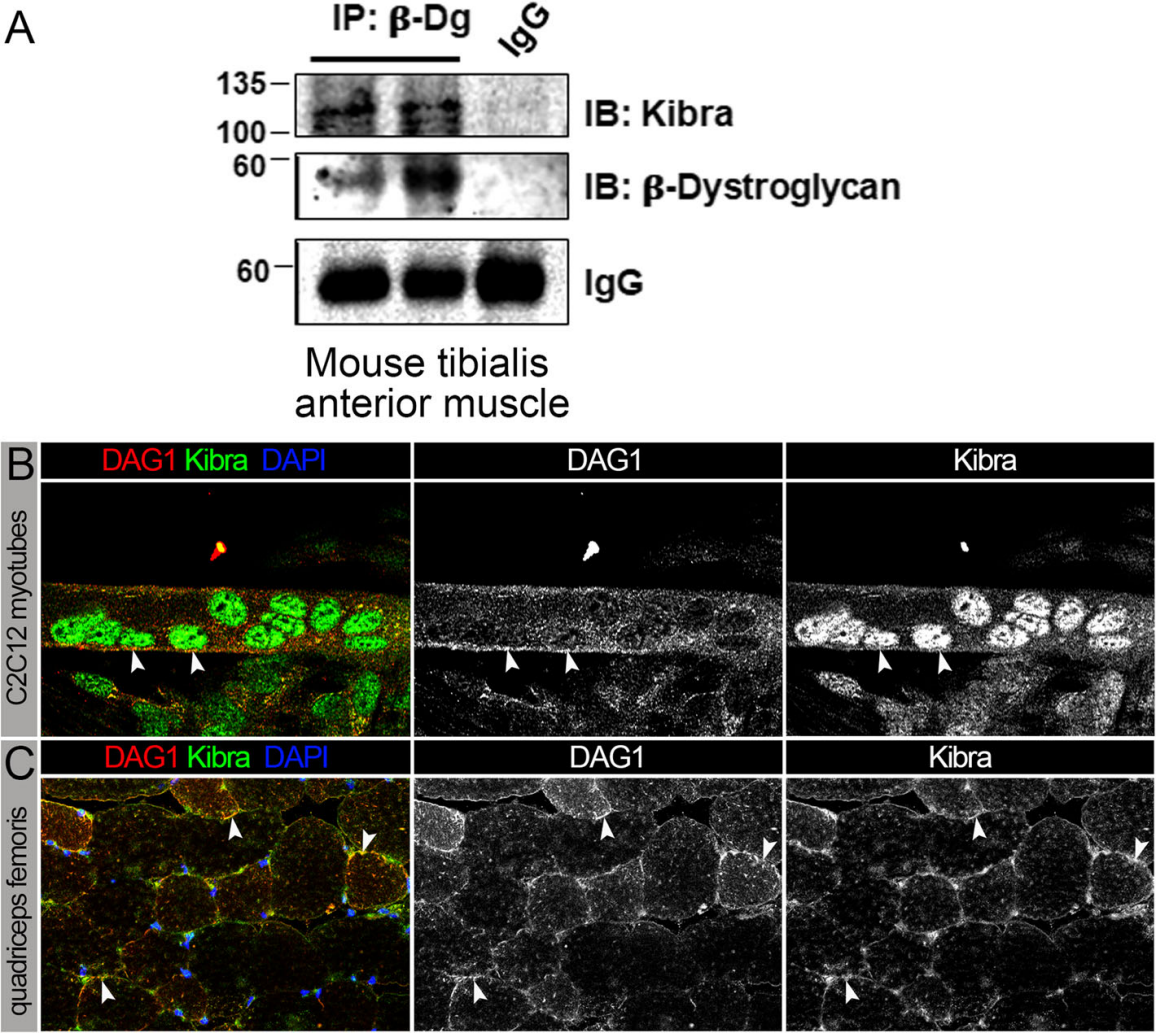
Similar to *Drosophila*, dystroglycan (DAG1) and Kibra interact in mammalian muscles. **a** Co-immunoprecipitation of beta-Dg (DAG1) and Kbr from mouse muscle extracts (*tibialis anterior* muscles, consisting of a mixture of oxidative and glycolytic muscle fibers) shows that the two proteins can form a complex in mammalian muscles. **b** Co-staining of mouse myotubes differentiated from C2C12 myocytes with anti-DAG1 (red) and anti-Kbr (green) antibodies. Arrowheads show the co-localization of both proteins at the sarcolemma of differentiated muscle cells in vitro. **c** Co-staining of the cryo-sections of mouse leg muscles (*quadriceps femoris*, a mixed slow- and fast-twitch oxido-glycolytic muscle) with anti-DAG1 and anti-Kbr antibodies demonstrate the co-localization of the proteins at the muscle sarcolemma in vivo, which is similar to our findings in *Drosophila* (see Additional file 2: Figure S2)

**Fig. 7:**
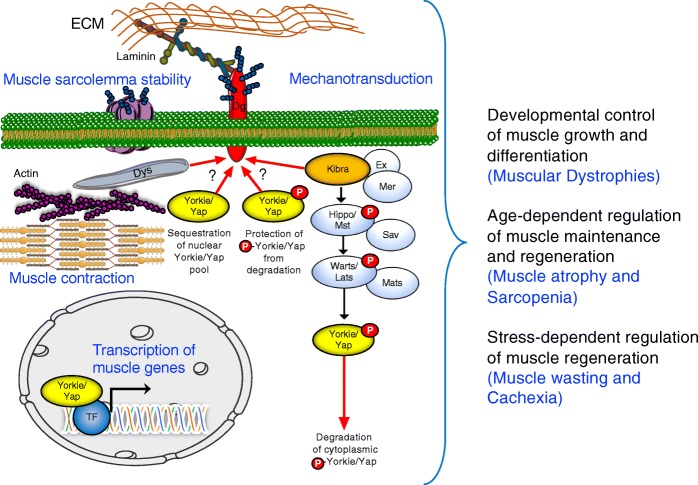
Dual roles of Dg as an ECM receptor for the dystrophin glycoprotein complex and a mechanotransducing receptor for Hippo signaling. Drawing of the DGC and the Hippo pathway association in the muscle cell. The DGC is positioned at the sarcolemma. The extracellular part of the transmembrane protein Dg binds the ECM protein LamininA, and Dg’s intracellular region interacts with the Hippo kinase signaling pathway components, Kbr and Yki. Kbr is a positive regulator of the Hippo kinase signaling that through the cascade of phosphorylation reactions results in phosphorylation of the transcriptional co-activator Yki. Phosphorylated Yki does not translocate to the nucleus to regulate gene expression but remains in the cytoplasm, where it is subjected to degradation or alternatively, it can be bound to Dg. Dg promotes Hippo signaling via association with Kbr and can interact with the Hippo transcriptional co-activator Yki. Hypothetically, Yki-Dg interaction can protect cytoplasmic phosphorylated Yki from degradation and contribute to the Yki pool for quick response to mechanical stress or Dg can sequester non-phosphorylated Yki preventing it from entering the nucleus for gene expression regulation. In adult *Drosophila* muscles, Hippo signaling is required to control muscle integrity and size, during development and upon aging. Our data show that Dg genetically interacts with Kbr and Yki to maintain muscle integrity during aging. Our results suggest that muscle Dg is a Hippo signaling receptor that transmits mechanical stress from the ECM to the cytosolic signaling regulating gene expression. However, it is not clear whether in adult muscles Dg-Laminin interactions influence Hippo signaling, whether Dg-Kbr/Yki and Dg-Dys complexes are related and if they rely on the interactions thought the same PPxY motifs on Dg’s C-terminus, whether Dg interacts with phosphorylated or non-phosphorylated Yki, and which genes are regulated by the Dg-Hippo pathway in adult muscles

In accordance with the studies in mammalian heart muscles [36, 78], our current work also demonstrates that in *Drosophila* muscles, Dg associates with Yki, suggesting that the previously proposed Dg function in Yki/Yap sequestration could be conserved. In addition, our data show that in *Drosophila*, Dg interacts with another Hippo pathway factor, Kbr, and recent studies demonstrated the possibility of Kbr-Dg interaction in human cells [74]. Therefore, we tested if Dg interacts with Kbr in mammalian muscles. Firstly, we immunoprecipitated beta-Dg (DAG1) from extracts from mouse tibialis anterior muscles, which consist of a mixture of oxidative and glycolytic muscle fibers. Then, we analyzed the protein precipitates by SDS-PAGE and immunoblotting using Kbr and beta-Dg antibodies. The co-immunoprecipitation assay shows that the two proteins also interact in mammalian muscles (Fig. 6a). Secondly, we tested Kbr-Dg co-localization using an immunohistochemical approach. For this, we co-stained mouse myotubes differentiated from C2C12 myocytes with anti-DAG1 and anti-Kbr antibodies (Fig. 6b). Analyses of staining patterns confirm the co-localization of both proteins at the sarcolemma of differentiated muscle cells in vitro. Thirdly, to confirm Dg-Kbr co-localization in vivo, we stained the cryo-sections of mouse leg muscles (Fig. 6c). We found that these two proteins co-localize at the muscle sarcolemma, which is similar to our findings in *Drosophila*. Together, these data for the first time show that the ECM receptor Dg interacts with the Hippo signaling component, Kbr in vivo; moreover, this interaction is evolutionarily conserved. Since in addition to Kbr, Dg also interacts with another Hippo component, Yki that plays an opposing to Kbr role in the transduction of Hippo signaling cascade, our data suggest that apart from being a physical link between the ECM and the cell cytoskeleton, muscle Dg functions as platform for Hippo cascade components to modulate the strength of this pathway (Fig. 7). Interestingly, Dg supports Hippo signaling and negatively regulates functional Yki, which can occur via two possibly synergistic mechanisms: (1) Dg binds Kbr at the sarcolemma, which stabilizes Kbr and its association with the associated proteins of the Hippo signaling cascade, which facilitates phosphorylation and degradation of cytoplasmic Yki; (2) Dg binds Yki at the sarcolemma, which sequesters it from the cytoplasmic pool and reduces the amount of Yki available for nuclear function. In the other model, Dg can bind the phosphorylated Yki and protect it from degradation. At least our genetic interaction data in IFMs propose that Dg and Kbr act together to enhance Hippo signaling, supporting the first model. However, more experiments are needed to dissect the exact mechanism of Dg-Yki and Dg-Kbr interaction.

## Discussion

### The ECM proteins

The majority of diagnosed secondary dystroglycanopathies are associated with abnormal glycosylation of Dg, which affects its association with the ECM molecules. One of the most important Dg glycosylases is LARGE [59, 79–86]. Intriguingly, while the *Drosophila* genome lacks LARGE homologs, it contains genes encoding *O*-mannosyltransferases, which are involved in the transfer of d-mannose from dolichyl-phosphate-d-mannose onto serine or threonine residues of target proteins, including Dg. These *O*-mannosyltransferases, Rt/POMT1 and tw/POMT2, function in association with each other to maintain normal muscle development and the sensory feedback controlling muscle contractions [87]. *POMT* genes are evolutionarily conserved in metazoans. Genetic defects in either protein *O*-mannosyltransferase, POMT1 or POMT2, underlie severe muscular dystrophies in humans. Specifically, a form of dystroglycanopathy, a limb-girdle muscular dystrophy (type C2; MDDGC2), is caused by homozygous or compound heterozygous mutation in the POMT2 gene. In addition, Walker-Warburg syndrome and muscle-eye-brain disease are also caused by genetic defects affecting these genes [88].

It is also noteworthy that the pair of threonines in α-Dg that are modified by LARGE is not conserved in multiple phyla [89]. In general, in some vertebrates, it was observed a lower degree of sequence conservation at the level of the N-terminal region of Dg, which normally consists of immunoglobulin-like domain G1 (IG1), S6 (similar to the Ribosomal protein S6, a constituent of ribosomes), and the heavily glycosylated mucin-like domains, which is of particular interest due to the importance of Dg’s binding to the ECM proteins, especially laminins, and also with regard to the general role of mucins that can sterically affect integrin clustering [90, 91]. For example, *Drosophila* Dg has a mucin-like domain that can play a role in Dg’s interaction with laminins; while *Caenorhabditis elegans* Dg completely lacks a mucin domain, and it is not clear whether or how it interacts with laminin. It was suggested, however, that the IG1 domain can show some weak laminin-binding activity and could provide an alternative mode of Dg-Laminin interaction or that binding to other ECM ligands such as perlecan has evolved to predominate over laminin binding [89]. Moreover, it has been previously found that levels of laminins as well as integrin depend on Dg expression levels [21]. Moreover, Dg is involved in the regulation of the ECM constitution and is capable of readjusting the levels of ECM components at the level of transcription [21]. Overexpression of a form of Dg containing only the extracellular, but lacking the intracellular, domains caused abnormal integrin and laminin distribution, demonstrating that the presence of the ECM-interacting domain of Dg is sufficient to modulate the expression of proteins.

In *Drosophila*, similar to vertebrates, Dg is also heavily glycosylated [50]. While Dg glycosylation has been intensively studied, some steps are still not very well understood. Importantly, we also found in our screen that *Drosophila* Dg, similar to mammalian Dg [92, 93], can interact with several lectin proteins, in particular with C-type lectins *CG6055*, *CG4115*, and *Clect27*. In general, Dg’s interactions with the ECM proteins, such as lectins and laminins, depend on elaborate carbohydrate modifications of Dg, which normally occur in the ER and Golgi [59, 88, 93]. We have identified several constituents of these subcellular organelles (Fig. 1d, Additional file 2: Table S1), which can be proposed as new candidates that could serve to manage proper Dg glycosylation. For example, among the detected proteins were a small GTPase Sar1 that is recruited to the endoplasmic reticulum and initiates the recruitment of the COPII subunit complex Sec23/Sec24 mediating anterograde protein transport from the ER to Golgi and two small GTPases that contribute to vesicle trafficking regulation, Rab7 and Rab18 (Additional file 2: Table S1). We did not expect to see Dg interaction with any modifying proteins per se because normally, most of the enzymatic interactions are transient, “kiss-and-run’ interactions, and thus unlikely to be detected by mass spectrometry. In general, identification of substrates of glycosylating enzymes is hampered by the weak affinity of substrates for the enzyme and because the complex between the substrate and the glycosylating enzyme is transient. However, we did identify an uncharacterized protein, encoded by *CG33303*, which could be involved in N-linked Dg glycosylation. *CG33303* is a homolog of human Riboporin 1, a subunit of oligosaccharyltransferase in the ER [94], and therefore, in the future, it would be interesting to study this protein in greater detail.

### Membrane-associated proteins

We detected seven membrane-associated factors that mainly mediate transmembrane transport (Fig. 1d, e; Additional file 2: Table S1): transmembrane ion channel components (PMCA, ATPalpha, nrv1, and Vha100-2) and a drug transporter (CG10226). It has been previously reported that besides providing structural integrity to the sarcolemma, Dg is also involved in the regulation of intracellular calcium ions (Ca^2+^), specifically handling the import/export of Ca^2+^ in response to the level of intracellular Ca^2+^ stored in the sarcoplasmic reticulum [20, 95]. Previously we reported that blockage of calcium channels rescues Dg-linked seizures in adult *Drosophila* [20]. Now, we identified that Dg associates with PMCA, a plasma membrane calcium ATPase (P-type ion pump) that functions as a low-capacity, high-affinity Ca^2+^ extrusion apparatus required for maintaining resting Ca^2+^ levels in all cells. It has been previously reported that in *Drosophila* larval NMJs, PMCA plays an important role in restoring resting Ca^2+^ levels after pre- or post-synaptic Ca^2+^ influx [96]. Our new findings suggest that Dg might associate with PMCA at the NMJ sites to regulate cellular Ca^2+^ levels, which is consistent with Dg protein elevation at the NMJ post-synapses [20]. In support of a role for Dg in the regulation of muscle cell calcium homeostasis, several calcium channel subunits (Cacna1c, Cacna1e, Cacnb3) were also co-purified with Dg in mice [9].

Another ion channel proteins that we identified to be putatively associated with Dg are - ATPα and Nrv1 (Nervana1), which are α- and β-subunits of the Na^+^, K^+^ ATPase ion pump important for maintaining cellular membrane potential in the muscles [97]. Previous studies report that the activity of α3 Na^+^, K^+^ ATPase can be modulated by the ECM protein Agrin in mouse cardiac myocytes, influencing their contraction [98]. The N-terminal region of Agrin binds laminin, while the C-terminal end binds to several transmembrane ECM receptors, including Dg; however, whether Agrin-linked regulation of Na^+^, K^+^ ion pump depends on Dg remains unrevealed.

Among identified proteins, we also detected a drug transporter of the ABC family. While drug transporters of the SLC family are largely uptake transporters that regulate the influx of small substances into the cell, the ABC family transporters function as efflux transporters (reviewed in [99]). Active efflux is a mechanism crucial for moving compounds like neurotransmitters, toxic substances, and antibiotics out of the cell. In fact, ABC transporters are handling diverse substances, including metabolites, antioxidants, signaling molecules, hormones, nutrients, and neurotransmitters [99]. Deeper studying of Dg interaction with these proteins may help to explain the impaired metabolism and increased sensitivity to metabolic stress observed in *Drosophila* Dg-deficient mutants [18, 39, 100]. Moreover, even though the molecular basis for Duchenne and Becker MDs has been known for several decades, until now, the only medications found to be effective in ameliorating the effects of these disorders are corticosteroids [101]. Recent work also showed a positive effect of glucocorticoid steroids and alendronate treatment on dystrophic phenotype in the LGMD mouse model [102]. A limited number of medications improving MDs demand a vast input into the research on new drug development and in-depth examination of drug response mechanisms in MD patients. Unusual drug reactions and complications during anesthesia were previously described for a high percentage of patients with MDs [103]. It was found that a polymorphism in genes encoding the ABC efflux drug transporters affect the handling of drugs in human cancers [104, 105]. Now, we identified the association of Dg with the ABC family transporters. Further investigation of the functional relevance for such interaction might be crucial for improving drug usage in MD treatment. Regarding the identified membrane-associated Dg-interacting proteins, the question of whether Dg regulates their function or ensures proper clustering and localization at the plasma membrane also remains open for further studies.

### Cytoskeletal proteins

It is well known that Dg is a linker between the ECM and the actin cytoskeleton. Similar to vertebrates, in *Drosophila*, the intracellular part of Dg binds the cytosolic protein Dys [13], which is connected to actin. Other studies also showed that the cytoplasmic tail of β-Dg interacts directly with F-actin [106]. We did not, however, detect Dys to associate with Dg in our proteomic experiments, and this may be explained by several factors. The mass spectrometric analysis of muscle proteins has detection limitations—in particular, molecules that are of relatively low abundancy or proteins with a large molecular mass that exists in weak association with a variety of other proteins [107, 108]. Indeed, these features describe Dys protein. First, the low abundance of Dg-Dys complexes is supported by the reported measurements; in mouse and rabbit muscles, Dg and Dys proteins are expressed with the ratio 41:1 and 43:1, respectively [9]. In *Drosophila* muscles, Dg levels detected by antibody staining are also noticeably higher in comparison with Dys levels [18]. Second, in the human genome, *dystrophin* (*DMD*) is the largest gene, spanning a genomic range above 2 Mb and encoding for a protein of 3685 amino acid residues [109]. Similarly, *Drosophila* Dys protein size can reach 3598 amino acids in length, depending on the isoform (annotated in *FlyBase*). Moreover, it has been shown that the Dg-Dys interaction is weak and occurs at the micromolar range [13], which adds a further challenge for protein complex detections using global proteomics analysis. Another possible explanation is that a direct interaction of Dg with Kbr or other Dg interactors could stably displace the interaction with Dys in the adult *Drosophila* muscles. Nevertheless, by our proteomics approach, we could detect several cytoskeletal proteins to interact with Dg (Additional file 2: Table S1), demonstrating the abundance or strong affinity of their interaction with Dg in muscles. In particular, we detected 16 components of the actomyosin cytoskeleton to be associated with Dg, among them myosins, myosin- and actin-binding proteins, and Ca^2+^-binding troponins (Fig. 1e, Additional file 2: Table S1). Therefore, it would be important to study further the molecular mechanisms of Dg’s association with these cytoskeletal proteins, as well as other cytosolic proteins regulating the cell cytoskeleton (Fig. 1e).

### Cytoplasmic proteins

Among the cytoplasmic proteins associated with Dg, we found factors that regulate protein biosynthesis, trafficking, and degradation, as well as components that control cellular metabolic processes and mitochondrial functions. The identification of ER-associated components, cytoplasmic vesicle-associated proteins, and a number of aminopeptidases that regulate protein degradation can help to follow Dg’s cellular lifecycle from biosynthesis in the ER to the degradation. Understanding of Dg’s cellular turnover can be useful for targeted manipulation of Dg protein levels by affecting its specific regulators. Together with Dg, we also co-purified proteins controlling cellular metabolic processes and factors associated with mitochondria (Fig. 1e, Additional file 2: Table S1).

### Nuclear proteins

Studies in mammalian system suggest that proteolysis, tyrosine phosphorylation, and translocation of Dg to the nucleus result in altered gene transcription [110]. A recent study in prostate cancer cells showed cell density-dependent γ-secretase and furin-mediated proteolysis of β-dystroglycan which could be stimulated by Notch, leading to nuclear targeting [111]. Another work uncovered the mechanism of retrograde trafficking of β-dystroglycan from the plasma membrane to the nucleus [112]. In immortalized mouse C2C12 myoblasts, after being synthesized in the ER, α-Dg and β-Dg are transported from the ER to the Golgi apparatus and then to the plasma membrane, to form the DGC. The phosphorylation on β-Dg on Tyr890 causes its endocytosis from the plasma membrane and retrograde intracellular trafficking to the ER and then to the nucleus, mediated by β-Dg interaction with the Sec61 translocon complex [112].

In the *Drosophila* model, nuclear functions of Dg have not yet been revealed. However, we found Dg to co-immunoprecipitate with four histone proteins that form a nucleosome complex (Fig. 1e, Additional file 2: Table S1). Further dissection of Dg nuclear functions and its association with nuclear proteins would reveal new regulatory mechanisms and show whether Dg’s role to regulate nuclear processes is evolutionarily conserved.

### Metabolic pathways

Importantly, in the Dg interactome, components from the glutathione metabolism-controlling pathway and oxidative phosphorylation have been identified. Previously, we reported that Dg-deficient mutants have impaired metabolism [39] and that their muscles are particularly sensitive to dietary restriction [18]. Additionally, we found that Dg genetically interacts with several mitochondrial proteins associated with NADH dehydrogenase activity used in the electron transport chain for the generation of ATP (Fig. 1d, Additional file 2: Table S1). Previously, it has been also shown that mutation in the mitochondrial gene encoding mitochondrial ribosomal protein LP34 significantly influences Dg phenotypes in *Drosophila* [113]. Unfortunately, to date, it is unclear how Dg influences metabolic processes in muscle cells. Depending on the metabolic activity, muscles can be classified into two major classes: type I, which are red in appearance, slow-twitching, highly oxidative, and fatigue-resistant; and type II, which are white, fast-twitching, glycolytic, and quickly fatigable [114]. Our data show that Dg interacts with cytoplasmic and mitochondrial proteins, which could potentially promote type I muscle fiber mode. Since it has been reported that in *Drosophila* flight muscles, glycolytic enzymes are co-localized along sarcomeres at M-lines and Z-discs and this co-localization is required for normal flight [115], it would be interesting in the future to test whether Dg could serve as a platform for localization of metabolic enzymes, and perhaps, mitochondria in the muscle cell.

### Dg and Hippo signaling

Our results demonstrate that the deregulation of Yki and Kbr results in muscle maintenance and regeneration defects resembling sarcopenia and cachexia, implying that proper functioning of the Hippo pathway in muscles is required to control muscle size and sustain muscle integrity in young and aged animals. Therefore, it is important to study in greater detail the up- and downstream regulators of this pathway, targeting of which could ameliorate muscle wasting phenotypes. In addition, our data show that Dg interacts with both Kbr and Yki, suggesting that these interactions could fine-tune the activity of Hippo signaling in the muscles, affecting age-dependent muscle maintenance and regeneration (Fig. 7).

The Hippo pathway is linked to the cellular signals originated from the extracellular environment and transduced inside the cell through the plasma membrane proteins. Therefore, the Hippo pathway scaffolding complex formed at the membrane may contribute to the activation of the pathway [28, 70, 116, 117]. Since we found that Kbr interacts with Dg, we propose that Dg acts as an additional transmembrane receptor for Hippo signaling. Hippo signaling has been also shown to regulate myogenesis, myocyte growth, and survival [32, 118–122]. In addition, it controls tissue homeostasis and regeneration, organ size, and stem cell self-renewal through the regulation of cell proliferation, cell communication, and apoptosis [68–70]. Interestingly, Yap-DAG1 interaction was enhanced by Hippo-induced Yap phosphorylation; however, Yap phosphorylation is not required for DAG1 binding in vitro, suggesting that Hippo phosphorylation of Yap promotes the Yap-DGC complex formation in vivo [36]. Yap was also co-immunoprecipitated with sarcoglycan complex protein, and Yap’s interaction with the Dg or Sgc was perturbed in *mdx* (Dys deficient) mice, suggesting that Dys is required for the Dg-Yap association. Interestingly, in vitro binding assays showed that to sequester Yap, Dg binds Yap’s WW domain by the C-terminal PPxY-motif [36]. Since Dg association with the Hippo serine/threonine protein kinase Lats2 (large tumor suppressor kinase 2) which is homologous to *Drosophila* Warts was not detected in the co-immunoprecipitation experiment, the authors suggested that other Hippo pathway components do not form a complex with the DGC, just phosphorylated Yap [36]. In our study, in addition to Yki/Yap, we co-immunoprecipitated together with Dg another WW domain-containing Hippo signaling protein, Kbr. Both proteins, Kbr and Yki, contain two WW binding sites that can potentially bind Dg’s PPxY motifs. It has also been previously established that Dg contains two conserved PPxY motifs at the C-terminal end; both of them are functional and can mediate interaction with Dys protein in mammals and flies [13, 19, 71, 72]. Intriguingly, it has been recently demonstrated that the WW domain of Kbr, but not YAP, can bind the beta-Dg peptide [74]; therefore, further in vivo studies are necessary to clarify the mode of Dg interaction with these two Hippo signaling components.

It has been previously shown in mammalian cell culture that YAP acts as a mechanotransducer, and its nuclear localization depends on basal integrin-Src signaling. Importantly, Src can directly phosphorylate YAP; however, it remains unclear whether integrin-Src signaling acts directly on YAP or via the canonical Hippo signaling pathway [123, 124]. In *Drosophila*, mechanical strain directly regulates the Hippo pathway and no evidence has been found for the involvement of other pathways, such as Src42A kinase, in the regulation of Yki [125]. This suggests that in different contexts, mechanosensing via the Hippo pathway can be employed to regulate either cell proliferation or cellular morphogenesis, or simply to help promote apical domain homeostasis. We think that the interaction of Dg with Yki also can be cell- and tissue-dependent. Therefore, it would be necessary to analyze Yki/Yap regulation by Dg and integrin/Src in different cell and tissue types in both vertebrate and invertebrate models to draw clear conclusions about the extent of Yki regulation by Dg or integrins. In addition, considering the importance of post-translational modifications of Dg for its proper functions, it would be important to study how abnormal Dg glycosylation would affect its interaction with Hippo signaling.

Together, our work in *Drosophila* adult muscles shows evolutionarily conserved relations between the DGC and Hippo kinase signaling and suggests that manipulations of the Hippo signaling pathway could impact the severity of muscular dystrophy, sarcopenia, and cachexia syndromes.

## Conclusions

Here, we used *Drosophila* as a genetic model to find new MD players. We identified the muscle-specific Dg complexome by mass spectrometry analysis. We identified Dg to be associated with two components of the recently discovered and conserved Hippo signaling pathway, the WW domain-containing proteins Kbr and Yki. We have shown that Dg is important for muscle maintenance and its functional association with Kbr and Yki is crucial to adjust Hippo signaling to influence muscle size and integrity during aging (Fig. 7). Based on our results, we propose that Dg transmits signals sensed by the extracellular region to the inside of the cell via interaction with multiple ECM, membrane-associated, and cytoskeletal proteins, including Hippo kinase signaling components, which regulate age-dependent muscle maintenance and regeneration. Importantly, among the identified Dg-interacting proteins were novel membrane-associated components, cytoskeletal proteins, and cytoplasmic and nuclear factors (Fig. 1, Additional file 2: Table S1). Therefore, systematic studies of Dg function, regulation, and association with other proteins are essential not only for understanding muscle cell function under constant mechanical stress, but also for uncovering novel markers for MD diagnostics and developing innovative possibilities for MD therapeutics.

## Supplementary information

**Additional file 1:** **Data S1.** Original mass spectrometry data of Dg interactors in muscles.

**Additional file 2:** **Table S1.** Muscle-specific Dg-interacting proteins. **Table S2.** Pathway enrichment in muscle-specific Dg-interacting components. **Table S3.** Human disease association enrichment analysis. **Table S4.** Dg interacts with the Hippo pathway causing age-dependent muscle degeneration. **Table S5.** Dg and Hippo pathway regulate muscle size during ageing. **Table S6.** Effect of Dg and Hippo deregulation on muscle size during ageing. **Figure S1.** Dystroglycan co-localizes with cytoskeletal proteins in muscles and associates with the WW domain protein Kbr. **Figure S2.** Kibra protein expression in muscles. **Figure S3**. Yorkie protein expression in muscles. **Figure S4.** The Hippo signaling pathway components Kibra and Yorkie are WW domain-containing proteins. **Figure S5**. Deregulation of Yorkie or Kibra affects muscle size. **Figure S6.** Deregulation of the Dg-Hippo signaling cascade affects muscle growth and maintenance.

## Data Availability

The authors declare that all data supporting the findings of this study are available within the article and its supplementary information files or from the corresponding author upon reasonable request.
