# Supplementary material for: Profiling of the muscle-specific dystroglycan interactome reveals the role of Hippo signaling in muscular dystrophy and age-dependent muscle atrophy
Source: BMC Med. 2020 Jan 21;18:8. doi: 10.1186/s12916-019-1478-3 (PMC6971923; doi:10.1186/s12916-019-1478-3)

Supplementary Information
Supplementary Tables

Supplementary Table 1. Muscle-specific Dg-interacting proteins

Gene Symbol	Protein name	FlyBase ID	Cellular localization	Molecular function	Human homolog(s)	Disease association/ Risk factors
Dg	Dystroglycan	FBgn0034072	Membrane-associated receptor	ECM receptor, Dystrophin-Glycoprotein complex component	DAG1	Muscular dystrophy-dystroglycanopathy
Atpα	Na pump α subunit	FBgn0002921	Membrane-associated	Metal ion binding, ATP binding, Cation transmembrane transporter, ATPase activity	ATP1A1-4	Alternating hemiplegia, Dystonia-12, Response to antipsychotic treatment, Migraine
PMCA	plasma membrane calcium ATPase	FBgn0259214	Membrane-associated	Calcium-transporting ATPase activity, Metal ion binding	ATP2B1-4	Spinocerebellar ataxia, Deafness, Coronary heart disease, Hypertension
nrv	nervana 1	FBgn0015776	Membrane-associated	Cation transporter, Protein binding, Sodium:Potassium-exchanging ATPase	ATP1B1	Hypertension
CG10226	CG10226	FBgn0035695	Membrane-associated	Drug transmembrane transporter, ATPase activity	ABCB1<br>ABCB4<br>ABCB5<br>ABCB11	Fasting plasma glucose, Metabolic syndrome, Cholestasis, progressive familial intrahepatic 2, Response to statin therapy, Gallbladder disease 1, Dental caries
Vha100-1	Vha100-1	FBgn0028671	Membrane-associated	Proton-transporting ATPase	ATP6V0A1	F-cell distribution, Renal tubular acidosis
LanA	Laminin A	FBgn0002526	ECM	Receptor binding	LAMA1-5	Colorectal cancer, Amyotrophic lateral sclerosis, Cardiomyopathy, Congenital muscular dystrophy, Type 2 diabetes
LanB1	Laminin B1	FBgn0261800	ECM	Receptor binding	LAMB1-4	Nephrotic syndrome, Pierson syndrome, IgG glycosylation, Ulcerative colitis, Lissencephaly 5, Epidermolysis bullosa
LanB2	Laminin B2	FBgn0267348	ECM	Receptor binding	LAMC1-3	Colorectal cancer, Cortical malformations, Coronary heart disease, Systemic lupus erythematosus, Epidermolysis bullosa
Cpr62Bc	Cpr62Bc	FBgn0035281	ECM	Component of chitin-based cuticle	ZNF160	No disease terms found
CG34034	CG34034	FBgn0054034	ECM	Unknown (involved in reproduction)	-	-

Supplementary Table 1. Muscle-specific Dg-interacting proteins *(continued)*

Gene Symbol	Protein name	FlyBase ID	Cellular localization	Molecular function	Human homolog(s)	Disease association/ Risk factors
wupA	wings up A	FBgn0283471	Cytoskeleton	Actin binding, Tropomyosin binding	TNNI1-3	Cardiomyopathy, Inflammatory bowel disease, Arthrogryposis
Gel	Gelsolin	FBgn0010225	Cytoskeleton	Actin binding, Actin filament polymerization, Calcium ion binding	GSN	Amyloidosis, Finnish type
Mlc-c	Myosin light chain cytoplasmic	FBgn0004687	Cytoskeleton	Myosin heavy chain binding, Calcium ion binding, Actin filament-based movement	MYL3	Cardiomyopathy
MIP21134p	Myosin alkali light chain 1	FBgn0002772	Cytoskeleton	Myosin heavy chain binding Calcium ion binding	MYL3	Cardiomyopathy
Msp300	Muscle-specific protein 300 kDa	FBgn0261836	Cytoskeleton	Cytoskeletal protein binding, Actin filament binding, Protein kinase binding	SYNE1	Autism spectrum disorder, Attention deficit-hyperactivity disorder, Bipolar disorder, Depression, Schizophrenia
zip	zipper	FBgn0265434	Cytoskeleton	Motor activity, ATPase activity, Myosin light chain binding	MYH10 MYH9 MYH11 MYH14	End-stage renal disease, Glomerulosclerosis, Optic disc size, Deafness, Peripheral neuropathy, Myopathy
Act42A	Actin 42A	FBgn0000043	Cytoskeleton	Structural constituent of cytoskeleton	ACTB ACTG1	Dystonia, juvenile-onset Deafness Myopathy
ck	crinkled	FBgn0000317	Cytoskeleton	Actin binding, Motor activity, Myosin light chain binding, Cadherin binding, ATPase activity	MYO7A	Deafness, Usher syndrome
sqh	spaghetti squash	FBgn0003514	Cytoskeleton	Myosin heavy chain binding, Calcium ion binding	MYL12B	Antineutrophil cytoplasmic antibody-associated vasculitis
tmod	Tropo-modulin	FBgn0082582	Cytoskeleton	Tropomyosin binding, Actin binding	TMOD1	Obesity-related traits
TpnC4	Troponin C isoform 4	FBgn0033027	Cytoskeleton	Calcium ion binding	CALM1	Ventricular tachycardia
TpnC47D	Troponin C at 47D	FBgn0010423	Cytoskeleton	Calcium ion binding	CALM1	Ventricular tachycardia
hts	hu li tai shao	FBgn0263391	Cytoskeleton	Actin binding	ADD1-3	Hypertension, Biliary atresia
CG3630	CG3630	FBgn0023540	Cytoskeleton	Actin binding	ABRA	Dental caries
αTub85E	α-Tubulin	FBgn0003886	Cytoskeleton	Microtubule cytoskeleton component	TUBA1A	Lissencephaly 3, Distal hereditary motor neuropathy type II
Chd64	Chd64	FBgn0035499	Cytoskeleton	Actin binding,	TAGLN3	No disease terms found

Supplementary Table 1. Muscle-specific Dg-interacting proteins *(continued)*

Gene Symbol	Protein name	FlyBase ID	Cellular localization	Molecular function	Human homolog(s)	Disease association/ Risk factors
S-Lap1	Sperm-Leucylaminopeptidase 1	FBgn0035915	Cytosol	Aminopeptidase, Metalloexopeptidase, Manganese ion binding, Proteolysis	-	-
S-Lap2	Sperm-Leucylaminopeptidase 2	FBgn0052351	Cytosol	Aminopeptidase, Metalloexopeptidase, Manganese ion binding, Proteolysis	LAP3	Tumor angiogenesis
S-Lap4	Sperm-Leucylaminopeptidase 4	FBgn0052064	Cytosol	Aminopeptidase, Metalloexopeptidase activity, Manganese ion binding, Proteolysis	LAP3	Tumor angiogenesis
S-Lap7	Sperm-Leucylaminopeptidase 4	FBgn0033868	Cytosol	Aminopeptidase, Metalloexopeptidase activity, Manganese ion binding, Proteolysis	LAP3	Tumor angiogenesis
Gpo-1	Glycerophosphate oxidase-1	FBgn0022160	Cytosol	Glycerol-3-phosphate dehydrogenase, Calcium ion binding	GPD2	Type 2 diabetes
CG6178	CG6178	FBgn0039156	Cytosol	Fatty-acyl-CoA synthase, Long-chain fatty acid-CoA ligase	ACSM3	Hypertension
CG10916	CG10916	FBgn0034312	Cytosol	Zinc ion binding, Ubiquitin-protein transferase	RNF2<br>BRCA1	Obesity-related traits, Breast-ovarian cancer susceptibility, Pancreatic cancer, susceptibility
gfzf	GST-containing FLYWCH zinc-finger protein	FBgn0250732	Cytosol	Glutathione transferase, Nucleic acid binding Ras protein signal transduction	GDAP1	Charcot-Marie-Tooth disease
CG33303	CG33303	FBgn0053303	Cytosol	Protein N-linked cocosylation	RPN1	No disease terms found
CG7970	CG7970	FBgn0035252	Cytosol	-	PXMP2	No disease terms found
eIF4E1	eukaryotic translation initiation factor 4E1	FBgn0015218	Cytosol	Translation initiation factor activity RNA cap binding	EIF4E	Autism 19 Pervasive developmental disorder
CG4115	CG4115	FBgn0038017	Cytosol	Carbohydrate binding	-	-
Clect27	C-type lectin 27kD	FBgn0031629	Cytosol	Carbohydrate binding	-	-
loopin-1	loopin-1	FBgn0259795	Cytosol	Aminopeptidase, metalloexopeptidase	LAP3	Tumor angiogenesis
kibra	kibra ortholog	FBgn0262127	Cytosol, Membrane	Protein binding	WWC1-3	Periodontal microbiota, Memory, Height
CG5554	CG5554	FBgn0034914	Cytosol	Protein disulfide isomerase activity	TMX1	No disease terms found

Supplementary Table 1. Muscle-specific Dg-interacting proteins *(continued)*

Gene Symbol	Protein name	FlyBase ID	Cellular localization	Molecular function	Human homologs	Disease association/ Risk factors
RpS27A	Ribosomal protein S27A	FBgn0003942	Cytosol	Structural constituent of ribosome	RpS27A	Inclusion body myositis
RpS10b	Ribosomal protein S10b	FBgn0285947	Cytosol	Structural constituent of ribosome	RPS10	Diamond-Blackfan Anemia
Cyt-b5	Cytochrome b5	FBgn0264294	Cytosol	Electron carrier, Heme binding	CYB5A	Methemoglobinemia, type IV
Sar1	Sar1 ortholog (S. cerevisiae)	FBgn0038947	Cytosol, Cytoplasmic vesicles, ER	GTPase activity, GTP binding, Scaffold protein binding	SAR1B	Chylomicron retention disease
Rab7 (*CG5915*)	Rab7 (CG5915)	FBgn0015795	Cytosol, Cytoplasmic vesicles, ER	Small GTPase, GTPase activity, GTP binding	RAB7A	Charcot-Marie-Tooth disease, type 2B
Rab18	Rab18	FBgn0015794	Cytosol, Cytoplasmic vesicles, ER	Small GTPase, GTPase activity, GTP binding	RAB18	Warburg Micro Syndrome
Surf4	Surfeit 4	FBgn0019925	Cytosol, Cytoplasmic vesicles, ER	unknown	SURF4	No disease terms found
CG33303	CG33303	FBgn0053303	Cytosol, ER	Protein N-linked glycosylation	RPN1	No disease terms found
Pmp70	Peroxisomal Membrane Protein	FBgn0031069	Cytosol, Membrane	ATPase activity ABC transporter type	ABCD3	Bile acid synthesis defect, Zellweger Syndrome
Flo-1	Flotillin 1	FBgn0024754	Cytosol, Membrane	Structural molecule activity, homologs regulate endocytosis	FLOT2	No disease terms found
Rpt1	Regulatory particle triple-A ATPase 1	FBgn0028687	Nucleus	ATPase activity, Proteasome 26S subunit component	PSMC2 PSMC5 PSMC6	Osteosarcoma
His4	Histone H4	FBgn0001200	Nucleus	Heterodimerization, DNA binding, Chromatin assembly/disassembly	-	-
His2A	Histone H2A	FBgn0001196	Nucleus	Heterodimerization, DNA binding, Chromatin assembly/disassembly	H2AFV	No disease terms found
His2B	Histone H2B	FBgn0001198	Nucleus	Heterodimerization, DNA binding, Chromatin assembly/disassembly	-	-
His2Av	Histone H2A variant	FBgn0001197	Nucleus	Heterodimerization, DNA binding, Chromatin assembly/disassembly	H2AFV H2AFZ	No disease terms found
yki	yorkie	FBgn0034970	Nucleus	Transcription factor binding, Protein binding, Transcription coactivator	YAP1	MRI atrophy measures, Polycystic ovary syndrome, Pubertal anthropometrics
baf	barrier to autointegration factor	FBgn0031977	Nucleus	DNA binding	BANF1	Nestor-Guillermo progeria syndrome
ben	bendless	FBgn0000173	Nucleus	Ubiquitin conjugating enzyme	UBE2N	Parkinson's disease
SMC1	Structural maintenance of chromosomes 1	FBgn0040283	Nucleus	Chromatin binding, DNA binding, ATP binding, Heterodimerization, Cohesin	SMC1A	Cornelia de Lange syndrome 2

Supplementary Table 1. Muscle-specific Dg-interacting proteins *(continued)*

Gene Symbol	Protein name	FlyBase ID	Cellular localization	Molecular function	Human homolog(s)	Disease association/ Risk factors
mRpS11	mitochondrial ribosomal protein S11	FBgn0038474	Mitochondria	Structural constituent of ribosome	MRPS11	No disease terms found
CG40042	Tim23, Translocase of inner mitochondrial membrane 23	FBgn0267976	Mitochondria	Mitochondrial inner membrane translocase subunit Tim17/Tim22/Tim23/ peroxisomal protein PMP24	TIMM23	No disease terms found
CG32230	ND-MLRQ, NADH dehydrogenase (ubiquinone) MLRQ subunit	FBgn0052230	Mitochondria	NADH dehydrogenase activity, Mitochondrial electron transport, NADH to ubiquinone	NDUFA4	No disease terms found
Tim17b	Tim17b	FBgn0263977	Mitochondria	Protein targeting to mitochondria	TIMM17B	No disease terms found
CG12400	ND-B14.5B, NADH dehydrogenase B14.5 B subunit	FBgn0031505	Mitochondria	NADH dehydrogenase, Mitochondrial electron transport	NDUFC2	No disease terms found
ox	oxen	FBgn0011227	Mitochondria	Ubiquinol-cytochrome-c reductase	UQCR10	No disease terms found
ScsβG	Succinyl-coenzyme A synthetase β subunit	FBgn0029118	Mitochondria	Succinate-CoA ligase (GDP-forming)	SUCLG2	No disease terms found
ttm50	tiny tim 50	FBgn0250874	Mitochondria	Protein tyrosine phosphatase	TIMM50	No disease terms found
CG14095	CG14095	FBgn0036870	unknown	unknown	-	-
CG34205	CG34205	FBgn0085234	unknown	unknown	POU2AF1	No disease terms found
CG34461	CG34461	FBgn0250833	unknown	unknown	ZNF160	No disease terms found
CG10373	Jwa ortholog	FBgn0032704	unknown	unknown	ARL6IP5	No disease terms found
CG5676	CG5676	FBgn0032200	unknown	unknown	FUNDC1-2	Personality dimensions
CG42323	CG42323	FBgn0259223	unknown	unknown	-	-
CG6055	CG6055	FBgn0031918	unknown	C-type lectin	CLEC3B	Inflammatory Diseases
CG13047	CG13047	FBgn0036594	unknown	unknown	-	-
mv	mauve	FBgn0265988	unknown	unknown	LYST	Chediak-Higashi syndrome
CG13335	CG42807	FBgn0261989	unknown	unknown	-	-
CG14946	CG14946	FBgn0032405	unknown	unknown	HSD17B11	Cutaneous T-cell lymphoma
CG10527	CG10527	FBgn0034583	unknown	Farnesoic acid O-methyl-transferase	-	-
CG1572	CG1572	FBgn0030309	unknown	unknown	MARVELD1	Facial paralysis, Facial nerve disease
CG13392	CG13392	FBgn0032033	unknown	unknown	TMEM126A TMEM126B	Height, Optic atrophy-7
CG4972	CG4972	FBgn0032217	unknown	unknown	NCLN	Hirschsprung Disease 1

Supplementary Table 2. Pathway enrichment in muscle-specific Dg-interacting components

KEGG Pathway ID	Pathway description	Count in gene set	Gene names	False discovery rate
04512	ECM Receptor interaction	4	Dg<br>LanA<br>LanB1<br>LanB2	4.25×10^{-5}
00480	Glutathione metabolism	5	Gfzf<br>S-Lap1<br>S-Lap2<br>S-Lap4<br>Loopin-1	0.0214
00190	Oxidative phosphorylation	4	CG32230<br>CG12400<br>Vha100-1<br>Vha100-2	0.0365

Supplementary Table 3. Human disease association enrichment analysis

Disease ID	Disease Categories	Count in gene set	P-value
MESH:D009369	Cancer	26	2.52×10^{-11}
MESH:D005767	Digestive system disease	13	2.11×10^{-8}
MESH:D002318	Cardiovascular disease	14	5.88×10^{-8}
MESH:D052801	Urogenital disease (male)	14	3.05×10^{-8}
MESH:D052776	Urogenital disease (female)	12	2.69×10^{-8}

Supplementary Table 4. Dg interacts with the Hippo pathway causing age-dependent muscle degeneration

Age	Genotype	Muscle degeneration			Statistics[2]			Total analyzed muscles[3]
		Strong, % (AVE±SD)[1]	Moderate, % (AVE±SD)[1]	Total, % (AVE±SD)[1]	Muscle degeneration compared to w^{1118}/+	Muscle degeneration compared to 7 day old	Genetic inter-action	
7-day old	w^{1118}/+	0.99±0.38	9.94±3.45	10.93±3.32	-	-	-	n=399
	Dg^{O55}/+	1.22±0.40	10.77±1.25	11.93±1.43	[a] p=0.053<br>[b] p=0.642	-	-	n=410
	yki^{B5}/+	0.87±0.35	10.73±1.41	11.60±1.37	[a] p=0.702<br>[b] p=0.763	-	-	n=458
	kbr^{del}/+	0.39±0.34	11.39±0.45	11.78±0.45	[a] p=0.114<br>[b] p=0.684	-	-	n=492
	Dg^{O55}/yki^{B5}	0.89±0.33	8.07±1.69	8.96±1.97	[a] p=0.744<br>[b] p=0.425	-	-	n=444
	Dg^{O55}/+,<br>kbr^{del}/+	0.73±0.72	10.72±1.33	11.44±1.24	[a] p=0.602<br>[b] p=0.815	-	-	n=429
	Mhc>Dg^{RNAi}	4.25±6.25	5.83±1.44	10.09±7.20	[a] p=0.429			n=423
	Mhc>yki^{RNAi}	0.68±0.68	17.65±6.16	18.32±5.49	[a] p=0.246	-	-	n=232
28-day old	w^{1118}/+	2.68±0.16	17.48±1.45	20.16±1.57	-	[a] p=0.002<br>[b] p=0.012	-	n=414
	Dg^{O55}/+	7.78±1.86	25.39±0.68	33.19±1.32	[a] p=0.009<br>[b] $p=3.9x10^{-4}$	[a] p=0.002<br>[b] $p=4.6x10^{-5}$	-	n=408
	yki^{B5}/+	7.11±0.28	19.47±1.89	26.58±2.16	[a] $p=1.9x10^{-5}$<br>[b] p=0.014	[a] $p=1.8x10^{-5}$<br>[b] $p=5.4x10^{-4}$	-	n=436
	kbr^{del}/+	6.82±1.72	25.98±1.55	32.80±1.32	[a] p=0.014<br>[b] $p=4.4x10^{-4}$	[a] p=0.003<br>[b] $p=1.3x10^{-5}$	-	n=439
	Dg^{O55}/yki^{B5}	6.07±1.66	17.06±0.47	23.14±1.69	[a] p=0.024<br>[b] p=0.089	[a] p=0.006<br>[b] $p=7.0x10^{-4}$	[c] p=0.298<br>[d] p=0.001<br>[e] p=0.349<br>[f] p=0.096	n=346
	Dg^{O55}/+,<br>kbr^{del}/+	11.55±2.07	38.34±5.20	49.90±4.05	[a] p=0.002<br>[b] $p=2.9x10^{-4}$	[a] p=0.001<br>[b] $p=9.5x10^{-5}$	[c] p=0.079<br>[d] p=0.002<br>[g] p=0.038<br>[h] p=0.002	n=438
	Mhc>Dg^{RNAi}	10.65±5.80	8.38±6.79	19.03±12.08	[a] p=0.032	-	-	n=484
	Mhc>yki^{RNA}	15.85±9.15	8.15±2.56	24.09±11.71	[a] p=0.045	-	-	n=319

[1]Average and SD are determined from 3 biological replicates; [2]Two-tailed Student's t-test was applied for statistical analysis; [3]in every biological replicate 105-168 muscles were analyzed; [a]statistical analysis of strong muscle degeneration phenotype; [b]statistical analysis of total muscle degeneration phenotype; [c]comparison of strong muscle degeneration phenotype to Dg^{O55}/+; [d]comparison of total muscle degeneration phenotype to Dg^{O55}/+; [e]comparison of strong muscle degeneration phenotype to yki^{B5}/+; [f]comparison of total muscle degeneration phenotype to yki^{B5}/+; [g]comparison of strong muscle degeneration phenotype to kbr^{del}/+; [h]comparison of total muscle degeneration phenotype to kbr^{del}/+

Supplementary Table 5. Dg and Hippo pathway regulate muscle size during ageing

Genotype	Age	Muscle #	Muscle size, (AVE±AD)[1] μ m²	Analyzed muscles, n	Correlation area, ρ	Average of relative muscle size, (AVE±AD)[1]	Average muscle atrophy (AVE±AD)[1] %	p-value[2]
Mhc-Gal4/+	Young 7-day old,	*1-2*	2503.4±91.9	30	0.98	1.00±0.00	0.0±3.8	-
		3-4	3225.0±73.8	36				
		5-6	3038.7±130.6	36				
		7-8	3003.6±70.6	34				
		9-10	2425.6±117.9	31				
		11-12	2132.8±118.8	28				
	Aged, 28-day old	*1-2*	2229.1±58.9	68		0.93±0.00	6.7±2.7	[3]p=0.0065
		3-4	2949.9±50.0	70				
		5-6	2773.8±82.3	70				
		7-8	2841.2±68.4	68				
		9-10	2305.6±63.3	62				
		11-12	2101.1±83.8	32				
Mhc>Dg^{RNAi}	Young 7-day old,	*1-2*	2071.9±108.9	36	0.98	0.81±0.04	18.6±3.7	[3]p=3.4E-12
		3-4	2364.9±43.4	46				
		5-6	2516.6±16.3	46				
		7-8	2531.6±22.8	46				
		9-10	1895.3±25.7	45				
		11-12	1851.0±54.8	36				
	Aged, 28-day old	*1-2*	1762.5±81.4	61		0.74±0.04	25.8±3.5	[3]p=2.6E-16 [4]p=0.00072
		3-4	2198.3±66.3	68				
		5-6	2340.1±137.8	65				
		7-8	2373.1±140.3	64				
		9-10	1782.0±127.4	53				
		11-12	1649.0±65.3	29				
Mhc>yki^{RNAi}	Young 7-day old,	*1-2*	1584.2±233.9	22	0.93	0.58±0.03	42.4±3.2	[3]p=2.4E-21
		3-4	1726.9±302.3	22				
		5-6	1730.7±250.0	22				
		7-8	1931.2±370.0	22				
		9-10	1452.5±189.9	22				
		11-12	1276.3±249.3	22				
	Aged, 28-day old	*1-2*	1187.5±229.3	22		0.47±0.03	53.1±2.6	[3]p=6.5E-26 [4]p=2.1E-07
		3-4	1467.6±294.7	21				
		5-6	1527.8±255.8	18				
		7-8	1523.3±273.3	16				
		9-10	1128.7±307.5	20				
		11-12	937.6±150.8	18				

Supplementary Table 5. Dg and Hippo pathway regulate muscle size during ageing *(continued)*

Genotype	Age	Muscle #	Muscle size, (AVE±AD)[1] μ m²	Analyzed muscles, n	Correlation area, ρ	Average of relative muscle size, (AVE±AD)[1]	Average muscle atrophy (AVE±AD)[1] %	p-value[2]
Mhc>kbr	Young 7-day old,	*1-2*	2364.0±86.3	60	0.98	0.93±0.04	6.6±4.0	[3]p=0.00010
		3-4	2907.3±63.2	65				
		5-6	2848.7±44.7	65				
		7-8	2935.5±29.6	64				
		9-10	2078.2±32.5	65				
		11-12	2100.8±45.0	58				
	Aged, 28-day old	*1-2*	2070.0±98.9	61		0.85±0.04	15.0±4.4	[3]p=3.2E-09 [4]p=0.00065
		3-4	2546.1±90.3	64				
		5-6	2580.1±84.9	64				
		7-8	2699.3±99.4	64				
		9-10	1949.4±87.4	63				
		11-12	1984.6±82.9	59				
Mhc>yki	Young 7-day old,	*1-2*	2501.5±46.4	42	0.99	0.98±0.05	1.9±4.5	[3]p=0.2667
		3-4	2953.7±54.6	51				
		5-6	3039.7±41.4	52				
		7-8	3005.8±57.9	52				
		9-10	2203.9±25.1	51				
		11-12	2259.3±67.6	50				
	Aged, 28-day old	*1-2*	2282.7±70.4	56		0.91±0.05	8.3±4.9	[3]p=9.6E-05 [4]p=0.0139
		3-4	2762.0±154.2	69				
		5-6	2837.7±171.0	71				
		7-8	2814.5±129.5	71				
		9-10	2050.8±59.1	69				
		11-12	2169.3±71.8	63				
Mhc>kbr^{RNAi}	Young 7-day old,	*1-2*	2519.3±61.9	48	0.97	0.98±0.04	1.6±4.0	[3]p=0.2538
		3-4	3067.4±62.8	69				
		5-6	3001.2±142.5	72				
		7-8	3039.5±107.6	66				
		9-10	2196.9±31.6	67				
		11-12	2212.2±65.7	49				
	Aged, 28-day old	*1-2*	2014.0±107.2	57		0.82±0.04	17.9±4.3	[3]p=5.4E-11 [4]p=7.9E-08
		3-4	2422.3±99.0	68				
		5-6	2530.3±133.4	75				
		7-8	2577.8±113.2	76				
		9-10	1896.9±86.0	76				
		11-12	1912.4±91.3	74				

[1] AVE±AD are determined from 3 biological replicates, each having at least 10 flies
[2] Two-tailed Student's t-test was applied for statistical analysis
[3] compared to 7-day old control, [4] compared to 7-day old animals of the same genotype

Supplementary Table 6. Effect of Dg and Hippo deregulation on muscle size during ageing

Genotype	Atrophy rate, % (AVE±AD)[1]	Pearson correlation	p-value[2]	Absolute atrophy, µm² (AVE±AD)	Normalized absolute atrophy (AVE±AD)[1]	Pearson correlation	p-value[2]
Mhc-Gal4/+	6.91±2.73	-	-	-188.09±85.11	1.00±0.45	-	-
Mhc>Dg^{RNAi}	8.51±2.85	0.265	0.062	-187.72±52.91	1.00±0.28	0.325	0.495
Mhc>yki^{RNAi}	19.89±5.16	-0.37	5.7xE-05	-321.55±70.13	1.71±0.37	-0.23	0.005
Mhc>kbr	14.37±2.33	0.827	1.6xE-08	-364.83±83.90	1.94±0.45	0.855	1.3xE-07
Mhc>yki	6.59±1.94	0.420	0.452	-175.35±52.65	0.93±0.28	0.540	0.306
Mhc>kbr^{RNAi}	16.73±2.86	0.762	5.3xE-09	-447.13±98.15	2.38±0.52	0.822	3.7xE-08

[1] AVE±AD are determined from all 12 muscles in each fly from 3 biological replicates each having at least 10 flies
[2] one-tailed Student's t-test was applied for statistical analysis.

Supplementary Figure Legends

Supplementary Figure 1. Dystroglycan co-localizes with cytoskeletal proteins in muscles and associates with the WW domain protein Kbr

A, Images showing protein expression patterns in larval body wall muscles for Dg in wildtype and Dg loss-of-function mutant (Dg^{O55}). **B**, Images showing protein expression patterns in larval body wall muscles for Dg and other cytoskeletal components such as Hts, WupA, and TpnC25D. Images are maximum intensity projections of multiple z-sections, allowing to better visualize muscle cell surface. Hts is enriched and co-localizes with Dg in NMJs, while WupA and TpnC25D co-localize with Dg in stripes. **C**, Co-immunoprecipitation of Hts with *Dg::GFP* analyzed via Western blotting using specific antibodies against Hts (Adducin). **D**, Images showing co-staining of anti-Dg and Hts, or WupA in rectal ampulla muscles. Dg and Hts co-localize at the muscle cell membrane, while Dg and WupA are located together at the membrane sites and in stripes. **E**, Images showing protein expression patterns in hindgut muscles for Dg and Hts, or WupA. Images show cross-section of hindgut muscles cells. Dg – red, Hts, WupA, TpnC25D– green, DAPI – blue. Expression patterns for Dg and Hts, WupA, and TpnC25D are shown in separate channels.

Supplementary Figure 2. Kibra protein expression in muscles

A-C, Kibra protein expression pattern detected by anti-Kibra antibodies in wild type muscles of larval body wall (**A**), adult rectal ampulla (**B**), and adult hindgut (**C**). **D**, Kibra protein expression pattern is detected by anti-Kbr antibodies in larval body wall muscles of animals that overexpress GFP-tagged Dg with the muscle driver (*mhc>Dg-GFP*). Dg-GFP expression is marked by green, Kibra by magenta, and Kibra and Dg co-localization by white rectangles. Kibra – magenta, DAPI – green (**A-D**), GFP (Dg)–green (**D**). Expression patterns for Dg and Kibra are shown in separate channels (**D**). Scale bar 10µm. **E-F,** The membrane-tagged GFP *(UAS-CD8::GFP)* and the full-length Dg tagged with GFP *(UAS-Dg::GFP)* are overexpressed using the muscle-specific *MHC-Gal4* driver to test the possibility of non-specific interaction between GFP and identified Dg interacting partners. **E,** Co-immunoprecipitation of Kbr with Dg::GFP using GFP-Trap beads. The presence of Kibra and Dg::GFP in the immune-complex is detected by western blotting using specific antibodies against Kibra and GFP. **F**, Immunoblotting with anti-GFP antibodies on the whole animal extracts, recognizes overexpression of GFP-tagged proteins: CD8::GFP and Dg::GFP, accordingly to the genotypes (*MHC>GFP* and *MHC>Dg::GFP*). Importantly, immunoblotting with anti-Kibra antibody

demonstrates that Kibra is detected only in *MHC>Dg::GFP* sample. Together, these data confirm the specificity of interaction between Kibra and Dg, and not Kibra and GFP.

Supplementary Figure 3. Yorkie protein expression in muscles

A-C, Expression pattern of Yki protein that is endogenously tagged by GFP in larval body wall muscles (**A**), adult rectal ampulla (**B**) and adult hindgut muscles (**C**). Yellow arrows point to gut muscles cells. (**D**) Co-localization of Yki (Yki::GFP) and Dg in larval body wall muscles. Yki::GFP expression is marked by green, Dg by red, and Yki and Dg co-localization by yellow rectangles, DAPI – blue. Expression patterns for Dg and Yki are shown in separate channels. Scale bar 10 μm.

Supplementary Figure 4. The Hippo signaling pathway components Kibra and Yorkie are WW domain-containing proteins

A, Protein domain architecture of *Drosophila* Dystrophin, Kibra and Yorkie proteins. Dys protein contains one WW domain, while Kibra and Yorkie possess two, which potentially can bind WW-binding sites at the Dg C-terminus. **B**, Sequence alignment of WW domains of the previously described Dg-binding partner Dystrophin, and newly identified Dg-interacting Hippo pathway components, Kibra and Yorkie. Conserved regions are color-coded.

Supplementary Figure 5. Deregulation of Yorkie or Kibra affects muscle size

A, Images of transverse sections of IFMs of 28-day old Control (*Mhc-Gal4/+*) muscles. **B**, Upon Yki downregulation using *Mef2-Gal4* driver (*Mef2>Yki^{RNAi}*), flies are born without IFMs. **C**, Upon Yki downregulation using *Mhc-Gal4* driver (*Mhc>Yki^{RNAi}*), IFM are formed; however, their size is significantly reduced (Supplementary Table 6). This phenotype is augmented by ageing; compare muscles in 7 day young and 28-day old *Mhc>Yki^{RNAi}* flies. **D**, Upon Kbr downregulation using *Mhc-Gal4* driver (*Mhc>Kbr^{RNAi}*), the size of IFMs is also notably reduced (Supplementary Table 6).

Supplementary Figure 6. Deregulation of the Dg-Hippo signaling cascade affects muscle growth and maintenance

Box plots show the muscle size distribution in 7-day young and 28-day old controls and mutants with muscle-specific up- and downregulation of *Dg*, *yki* and *kbr.* Numbers correspond to muscle pairs (see Figure 5).

Supplementary Figures

Supplementary Figure 1

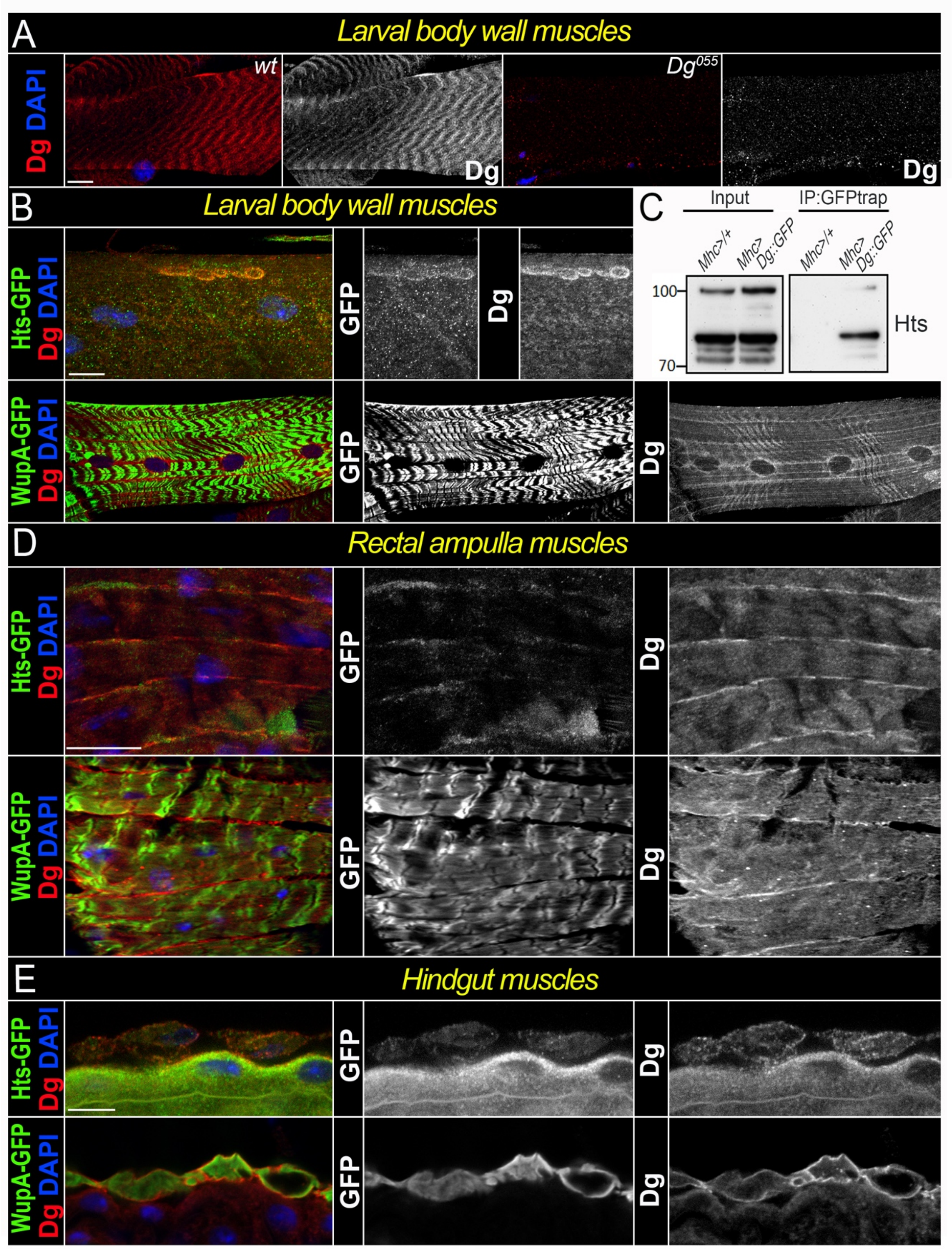

Supplementary Figure 2

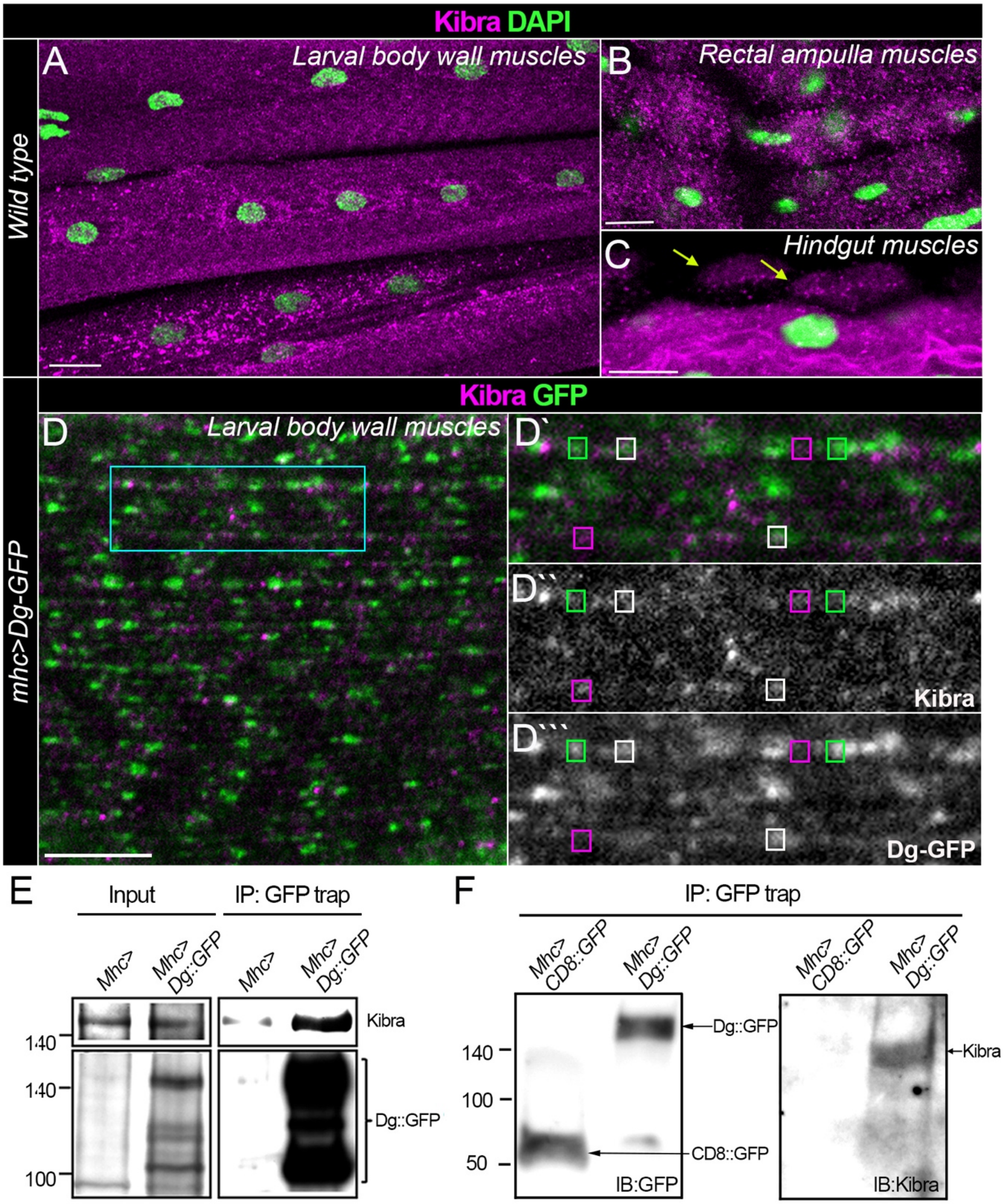

Supplementary Figure 3

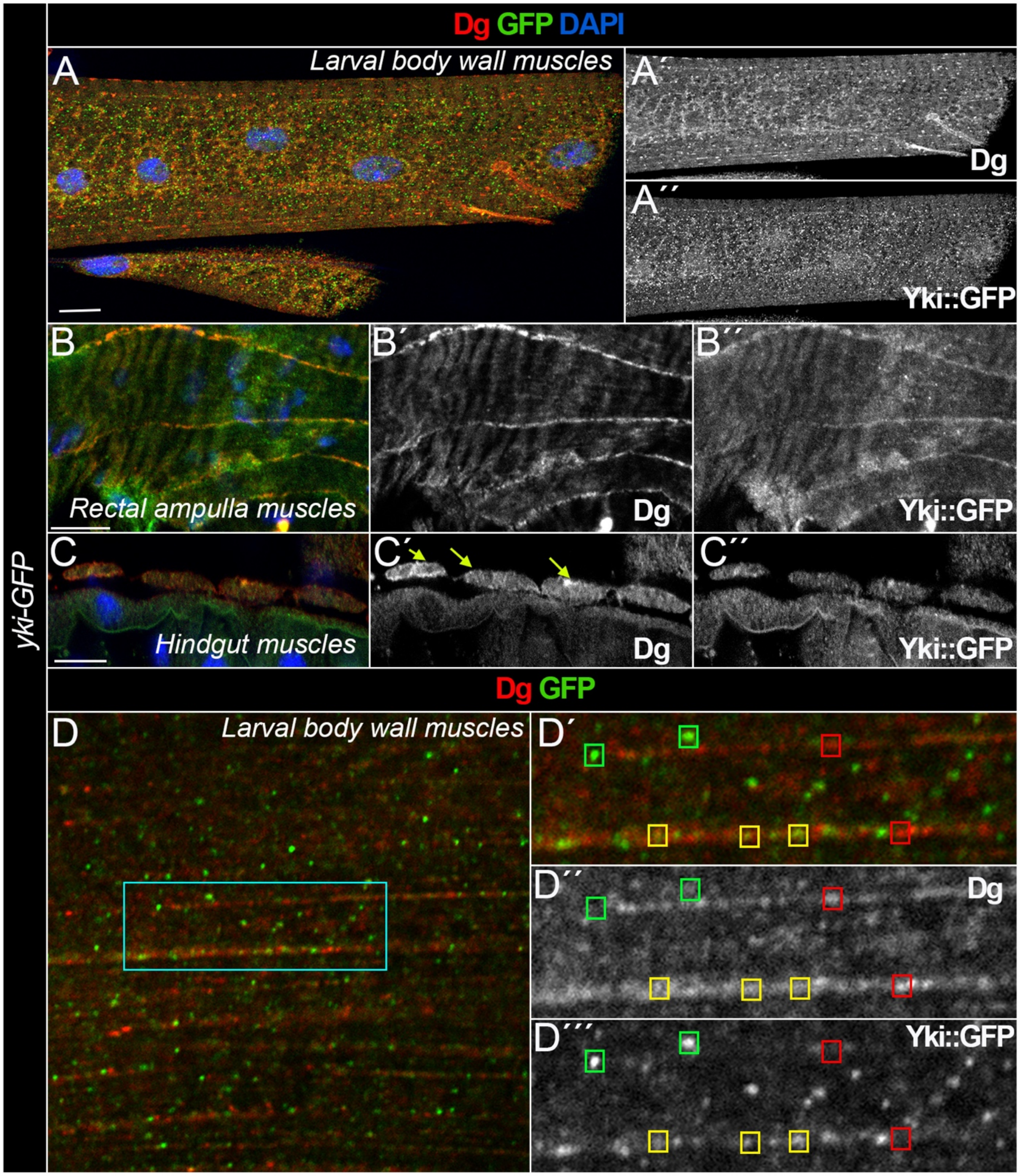

Supplementary Figure 4

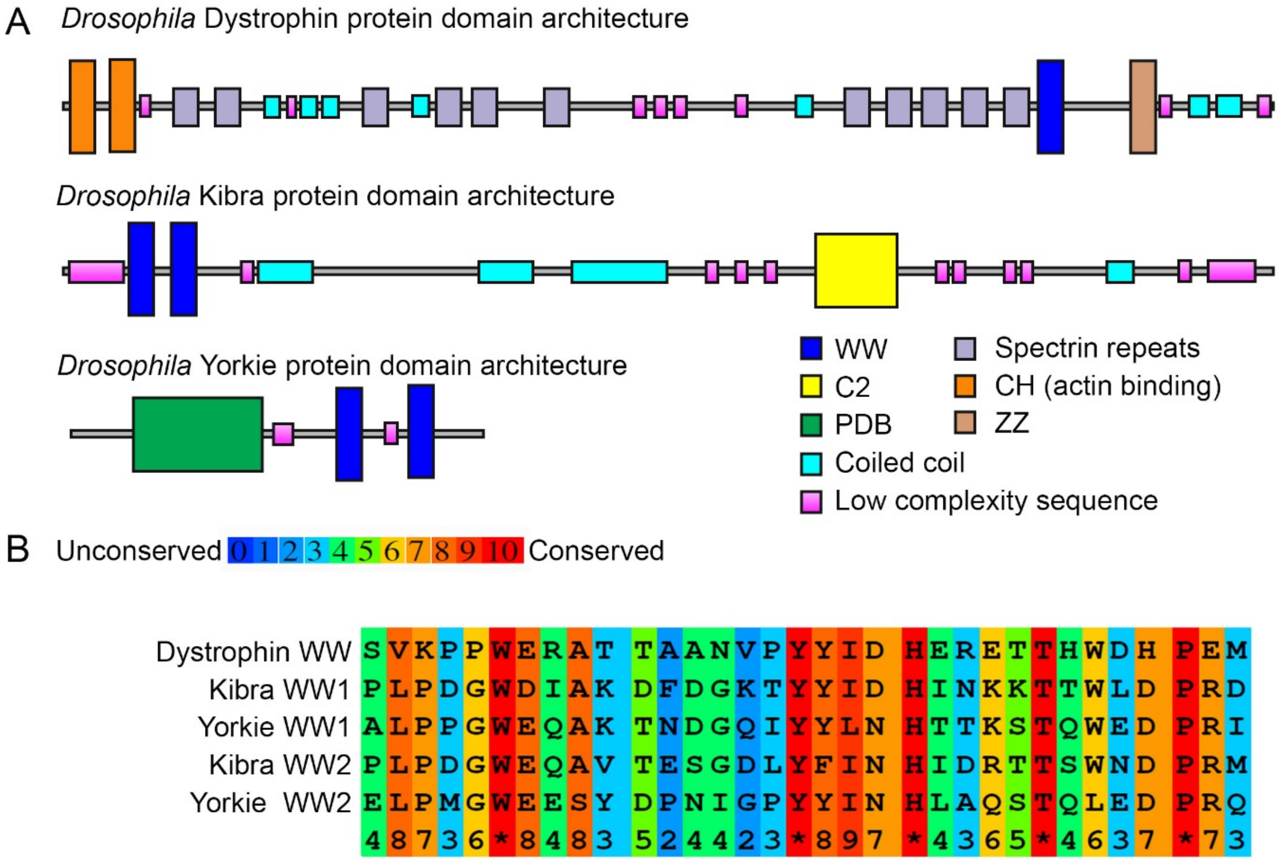

Supplementary Figure 5

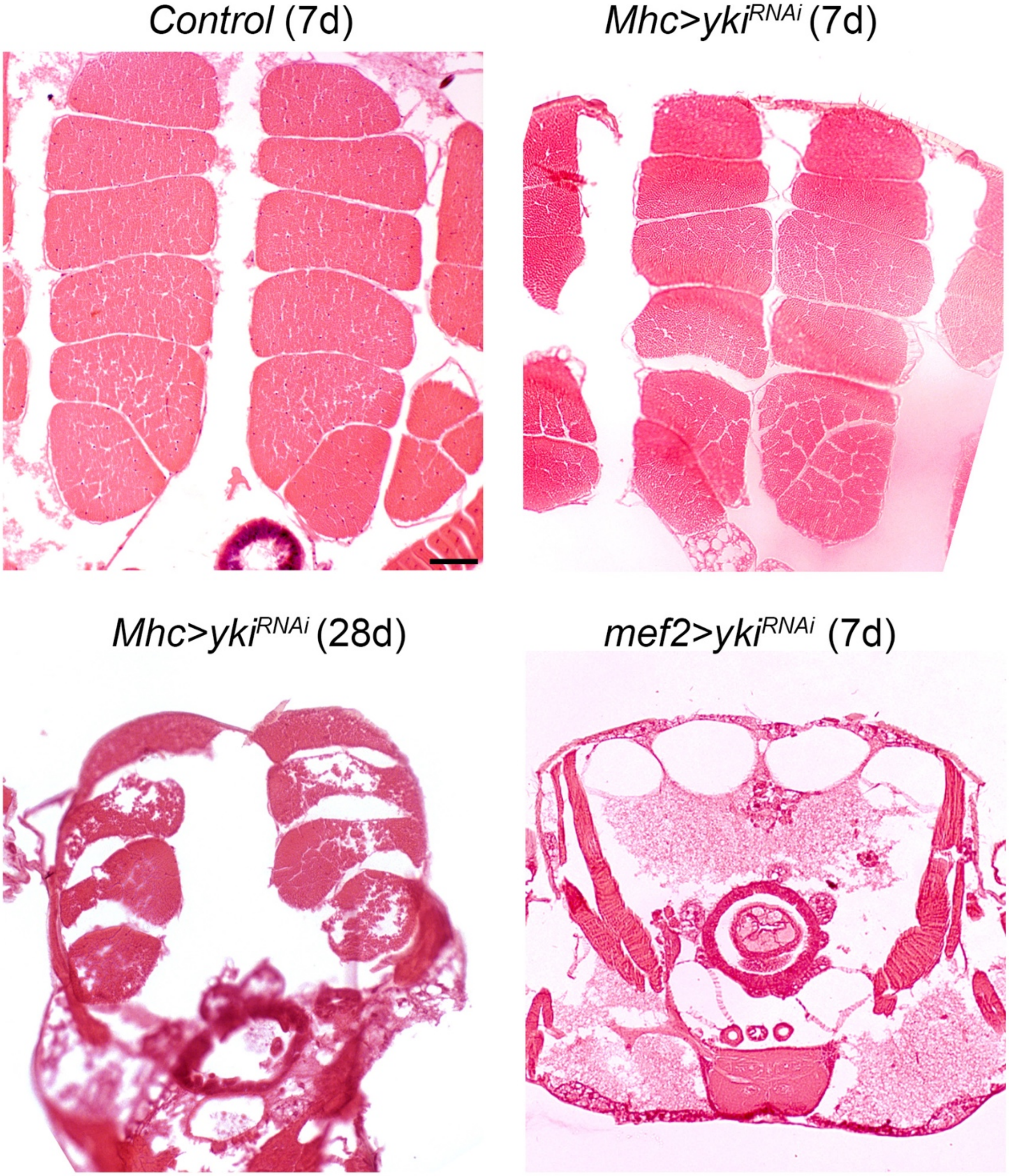

Supplementary Figure 6

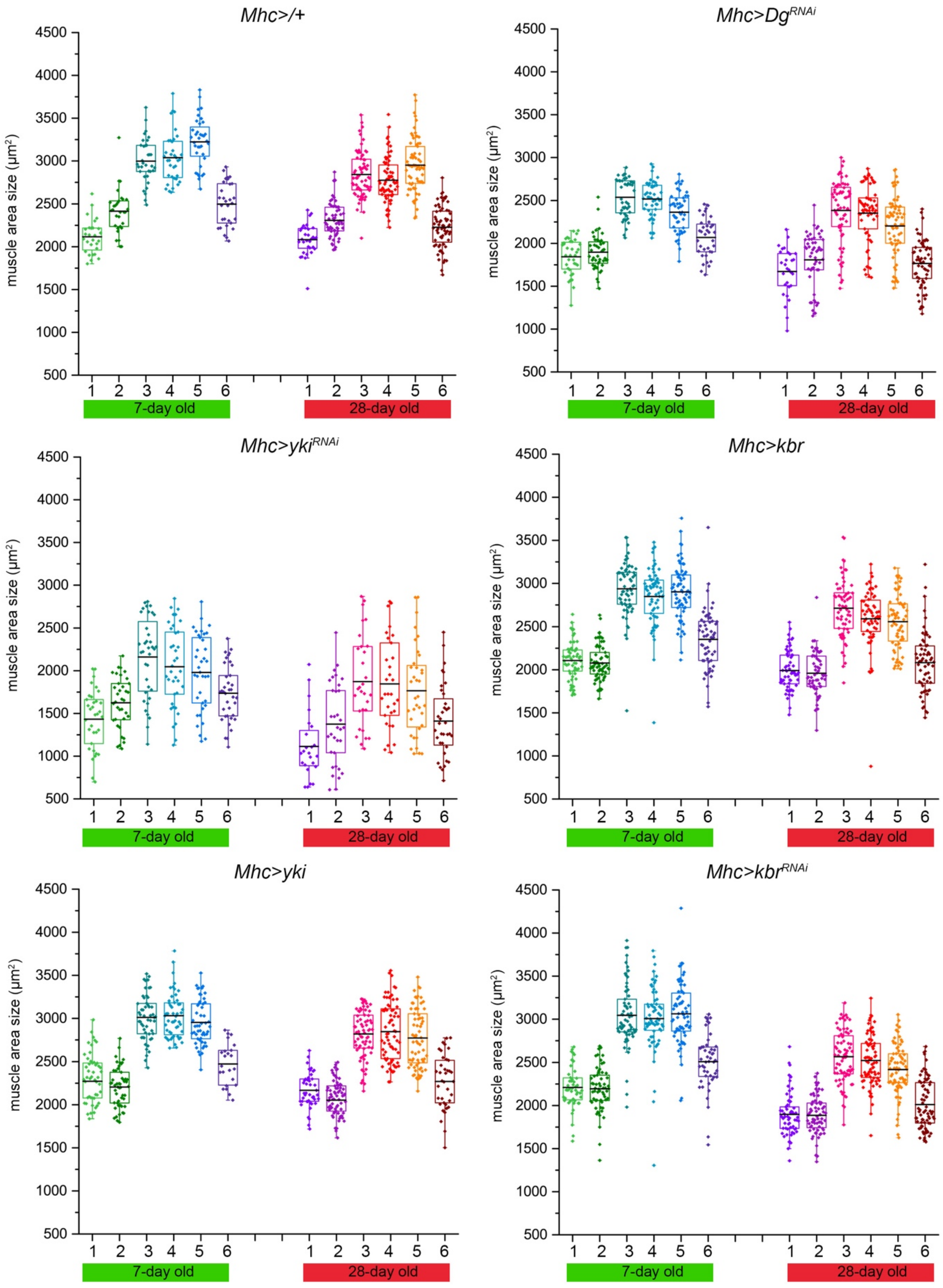

Supplement: Supplementary file 2 — Additional file 2: Table S1. Muscle-specific Dg-interacting proteins. Table S2. Pathway enrichment in muscle-specific Dg-interacting components. Table S3. Human disease association enrichment analysis. Table S4. Dg interacts with the Hippo pathway causing age-dependent muscle degeneration. Table S5. Dg and Hippo pathway regulate muscle size during ageing. Table S6. Effect of Dg and Hippo deregulation on muscle size during ageing. Figure S1. Dystroglycan co-localizes with cytoskeletal proteins in muscles and associates with the WW domain protein Kbr. Figure S2. Kibra protein expression in muscles. Figure S3. Yorkie protein expression in muscles. Figure S4. The Hippo signaling pathway components Kibra and Yorkie are WW domain-containing proteins. Figure S5. Deregulation of Yorkie or Kibra affects muscle size. Figure S6. Deregulation of the Dg-Hippo signaling cascade affects muscle growth and maintenance. [file 12916_2019_1478_MOESM2_ESM.pdf]
